# Structural adaptation associated with signaling preference at the human Growth Hormone–Releasing Hormone Receptor

**DOI:** 10.1093/procel/pwag016

**Published:** 2026-03-18

**Authors:** Ming-Wei Wang, Kaini Hang, Yue Qiu, Xianyue Chen, Yanyan Chen, Shengtian Huang, Zhaotong Cong, Qingtong Zhou

**Affiliations:** Department of Pharmacology, School of Basic Medical Sciences, Fudan University, Shanghai 200032, China; Department of Biology, Research Center for Deepsea Bioresources, Sanya 572025, China; Research Center for Medicinal Structural Biology, National Research Center for Translational Medicine at Shanghai, State Key Laboratory of Medical Genomics, Ruijin Hospital, Shanghai Jiao Tong University School of Medicine, Shanghai 200025, China; Engineering Research Center of Tropical Medicine Innovation and Transformation of Ministry of Education, School of Pharmacy, Hainan Medical University, Haikou 570228, China; Department of Chemistry, School of Science, The University of Tokyo, Tokyo 113-0033, Japan; Department of Pharmacology, School of Basic Medical Sciences, Fudan University, Shanghai 200032, China; Jinhua Key laboratory of Quality Evaluation and Standard Research of Traditional Chinese Medicine, Jinhua Institute for Food and Drug Control, Jinhua Advanced Research Institute of Technology, Jinhua 321019, China; Department of Biology, Research Center for Deepsea Bioresources, Sanya 572025, China; Department of Biology, Research Center for Deepsea Bioresources, Sanya 572025, China; Department of Pharmacology, School of Basic Medical Sciences, Fudan University, Shanghai 200032, China; Department of Pharmacology, School of Basic Medical Sciences, Fudan University, Shanghai 200032, China; Institute of Herbgenomics, Chengdu University of Traditional Chinese Medicine, Chengdu 611137, China; Research Center for Medicinal Structural Biology, National Research Center for Translational Medicine at Shanghai, State Key Laboratory of Medical Genomics, Ruijin Hospital, Shanghai Jiao Tong University School of Medicine, Shanghai 200025, China

**Dear Editor,** 

Growth hormone-releasing hormone (GHRH) receptor (GHRHR) belongs to class B1 G protein-coupled receptors (GPCRs), and is mainly expressed in growth-stimulating somatotropic cells of the anterior pituitary gland (Rivier et al., 1982; Guillemin et al., 1982; Mayo et al., 2003). GHRH secreted from the hypothalamus binds and activates GHRHR, evoking G_s_-coupled cAMP signaling and stimulating growth hormone (GH) secretion, which governs systemic growth and lipid and protein metabolism (Granata et al., 2025). Consequently, either insufficient or excessive GHRHR signaling is associated with diseases: isolated GH deficiency and dwarfism when the signal is too low; gigantism, acromegaly, lipodystrophy, and certain cancers upon abnormally high stimulation (Corazzini and Salvatori, 2013). GHRHR is therefore a clinically validated target whose agonists are sought for GH deficiency syndromes, whereas antagonists may restrain hormone-dependent tumors (Mayo et al., 2003; Halmos et al., 2025). The representative antagonist, MIA-602, a synthetic 30‑amino acid peptide (Zarandi et al., 2017), has demonstrated potent antitumor activity and exerts broad antioxidative benefits (Perez et al., 2012; Zhang et al., 2020). PCO371, an allosteric agonist originally developed for parathyroid hormone 1 receptor (PTH1R), also potently activates GHRHR (EC_50_ within one order of magnitude of that for PTH1R) (Zhao et al., 2023; Kobayashi et al., 2023). We have previously reported the cryogenic electron microscopy (cryo-EM) structures of GHRHR–G_s_ and splice variant 1 (SV1)–G_s_ complexes bound by GHRH, revealing the molecular basis of peptide binding, receptor activation, and SV1-mediated biased signaling (Cong et al., 2021; Zhou et al., 2020).

To elucidate the structural basis for its conformational plasticity and ligand-dependent signaling, we studied three different functional states of GHRHR, the ligand-free and G_s_-coupled (*Apo*), the allosteric agonist PCO371-bound and the antagonist MIA-602-bound, using cryo-EM and molecular dynamics (MD) simulations. The *Apo* GHRHR–G_s_ and PCO371–GHRHR–G_s_ cryo-EM structures were determined with global resolutions of 3.04 Å and 2.88 Å, respectively (Figs. 1A, 1B, S1 and S2). The high-resolution cryo-EM maps are sufficiently clear to allow for structural modelling of the receptor, the G_s_ heterotrimer, and the bound small-molecule agonist PCO371 (Fig. S3 and Table S1).

**Figure 1.**
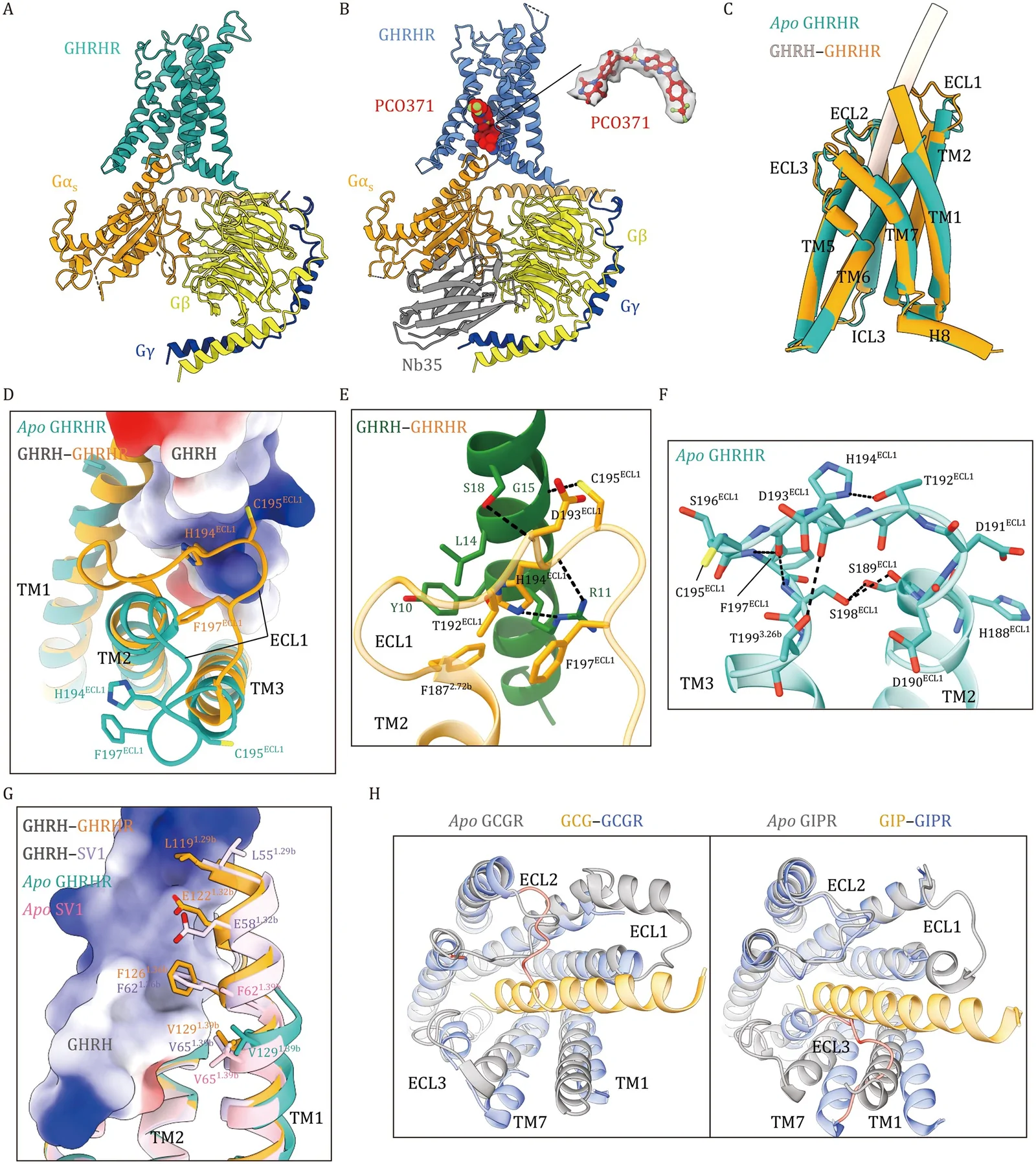
Cryo-EM structure of the Apo and PCO371-bound GHRHR–G_s_ complexes. (A) Cryo-EM structure model of the *Apo* GHRHR–G_s_ complex. Light sea green, *Apo* GHRHR; orange, Gα_s_; yellow, Gβ1; blue, Gγ2. (B) Cryo-EM structure model of the PCO371–GHRHR–G_s_ complex. Cornflower blue, PCO371-bound GHRHR; red, PCO371; orange, Gα_s_; yellow, Gβ1; blue, Gγ2; gray, Nb35. The bound PCO371 is detailed in separate close-up views, with the experimental EM density displayed in surface representation. (C) Superimposition of *Apo* and GHRH-bound GHRHR–G_s_ (PDB: 7CZ5) complexes from an orthogonal view, aligned on the receptor. (D) Superposition of *Apo* GHRHR and GHRH-bound GHRHR, highlighting the inward folding of ECL1 upon peptide binding. GHRH is shown in surface representation, and key ECL1 residues are shown as sticks. (E) Detailed view of interactions between the inward-folded ECL1 and the central region of GHRH (residues 10–18) in the GHRH–GHRHR complex. The polar contacts are shown as black dashed lines. Key receptor residues are shown as sticks and labeled with the class B1 GPCR numbering in superscript (Wootten et al., 2013). (F) Detailed view of the intramolecular polar network stabilizing the conformation of ECL1 in *Apo* GHRHR. (G) Conformational comparison of the extracellular half of TM1 across the four determined G_s_-coupled GHRHR structures, demonstrating its disorder in the *Apo* state and helix formation upon GHRH binding. (H) Comparison of *Apo* and peptide-bound structures reveals that, in the absence of peptide, the orthosteric pocket is partially occupied by distinct inward-folded segments: ECL2 in GCGR and the TM6–ECL3 region in GIPR.

Structural superposition reveals that the transmembrane domain (TMD) of the *Apo* GHRHR aligns well with that of the GHRH-bound GHRHR (PDB: 7CZ5), showing a low Cα root-mean-square deviation (RMSD) of 1.01 Å (Figs. 1C and S4A). This structural similarity suggests that the *Apo* receptor can adopt a conformation compatible with G_s_ coupling, which is supported by functional studies showing low-level basal G_s_ activity in the absence of agonist using a sensitive NanoBiT assay (Cong et al., 2021; Zhou et al., 2020). While the intracellular region of GHRHR adopts a conserved architecture upon G_s_ coupling, the extracellular halves exhibit a pronounced structural adaptability, primarily dictated by the absence of bound peptide agonist and the integrity of extracellular domain (ECD; Figs. 1D–G and S4). This plasticity is most evident in extracellular loop 1 (ECL1) and the extracellular half of TM1, with subtle variations in ECL3 and TM7, whereas ECL2 and TMs 2–5 remain largely rigid. In GHRH-bound structures (both GHRHR and SV1), ECL1 folds inward toward the orthosteric pocket to engage the middle region (residues 10–18) of GHRH, which is stabilized by specific interactions, including hydrogen bonds between R11^GHRH^ and the backbone of H194^ECL1^, and between S18^GHRH^ and D193^ECL1^, as well as stacking interactions between Y10^GHRH^ and F187^2.72b^ [class B1 GPCR numbering in superscript (Wootten et al., 2013)] (Fig. 1E). In contrast, in the *Apo* GHRHR, ECL1 adopts a naturally extended conformation along TM2 and TM3, stabilized by an intramolecular polar network involving TM2–ECL1–TM3 segments (e.g., hydrogen bonds between sidechains of T192^ECL1^ and H194^ECL1^; backbone atoms between H194^ECL1^ and F197^ECL1^/S198^ECL1^; and sidechain of S198^ECL1^ and the backbone/sidechain atoms of S189^ECL1^) (Fig. 1F). This results in a substantial outward displacement of ECL1 in *Apo* GHRHR, with the Cα atoms of C195^ECL1^ moving by 16.7 Å and 16.2 Å relative to its position in GHRH-bound GHRHR and SV1, respectively (Figs. 1D and S4A). A similar outward movement of ECL1 is observed in the *Apo* SV1 structure, further validating the adaptability of this loop in response to ligand binding (Fig. S4B). Notably, the integrity of the ECD significantly influences ECL1 dynamics (Fig. S4E). The extracellular half of TM1 represents another key element of the peptide-binding pocket with marked adaptability (Fig. 1G). In GHRH-bound structures, this segment forms an extended α-helix (8 residues) that shapes the binding pocket through hydrophobic contacts (e.g., L119^1.29b^ and E123^1.33b^) and stacking interactions (especially F126^1.36b^). In the *Apo* GHRHR, however, this region is disordered and could not be modeled, underscoring its role in ligand-induced structural reorganization (Fig. 1G). Similarly, ECL3 (e.g., D350^ECL3^) and the extracellular ends of TM7 (e.g., L354^7.35b^) slightly shift inward upon GHRH binding but adopt outward positions in the *Apo* GHRHR and SV1 structures (Fig. S4F).

The extracellular plasticity in the *Apo* state of GHRHR contrasts with observations in other class B1 GPCRs. For instance, in the *Apo* structures of the glucagon receptor (GCGR) and glucose-dependent insulinotropic polypeptide receptor (GIPR), the orthosteric peptide-binding pocket is partially occupied by the inward-folded receptor segments, namely, ECL2 in GCGR and the TM6–ECL3 junction in GIPR (Cong et al., 2024) (Fig. 1H). In GHRHR, however, the corresponding regions (ECL2 and ECL3) remain outward-facing, resulting in a more open extracellular vestibule in the absence of ligand. Collectively, these atomic-level observations demonstrate that the structural adaptability of the GHRHR extracellular domain, governed by agonist occupancy and ECD integrity, enables selective engagement of its cognate peptide.

The cryo-EM structure of the PCO371–GHRHR–G_s_ complex definitively resolves the binding mode of this synthetic agonist (Figs. 1B and S3B). PCO371 binds to a well-defined, predominantly hydrophobic pocket located deep within the intracellular half of the TMD, spatially distinct from the orthosteric pocket, with the closest atom of PCO371 positioned approximately 11.2 Å from the bound GHRH (Fig. 2A). Several key residues (F338^6.49b^ on TM6 and Y376^7.57b^ on TM7) act as gatekeepers, shaping and enclosing the pocket by forming a constricted region that sequesters the ligand from the lipid bilayer (Fig. 2A). This binding mode unequivocally classifies PCO371 as an allosteric agonist that modulates receptor activity from a unique intracellular site.

**Figure 2.**
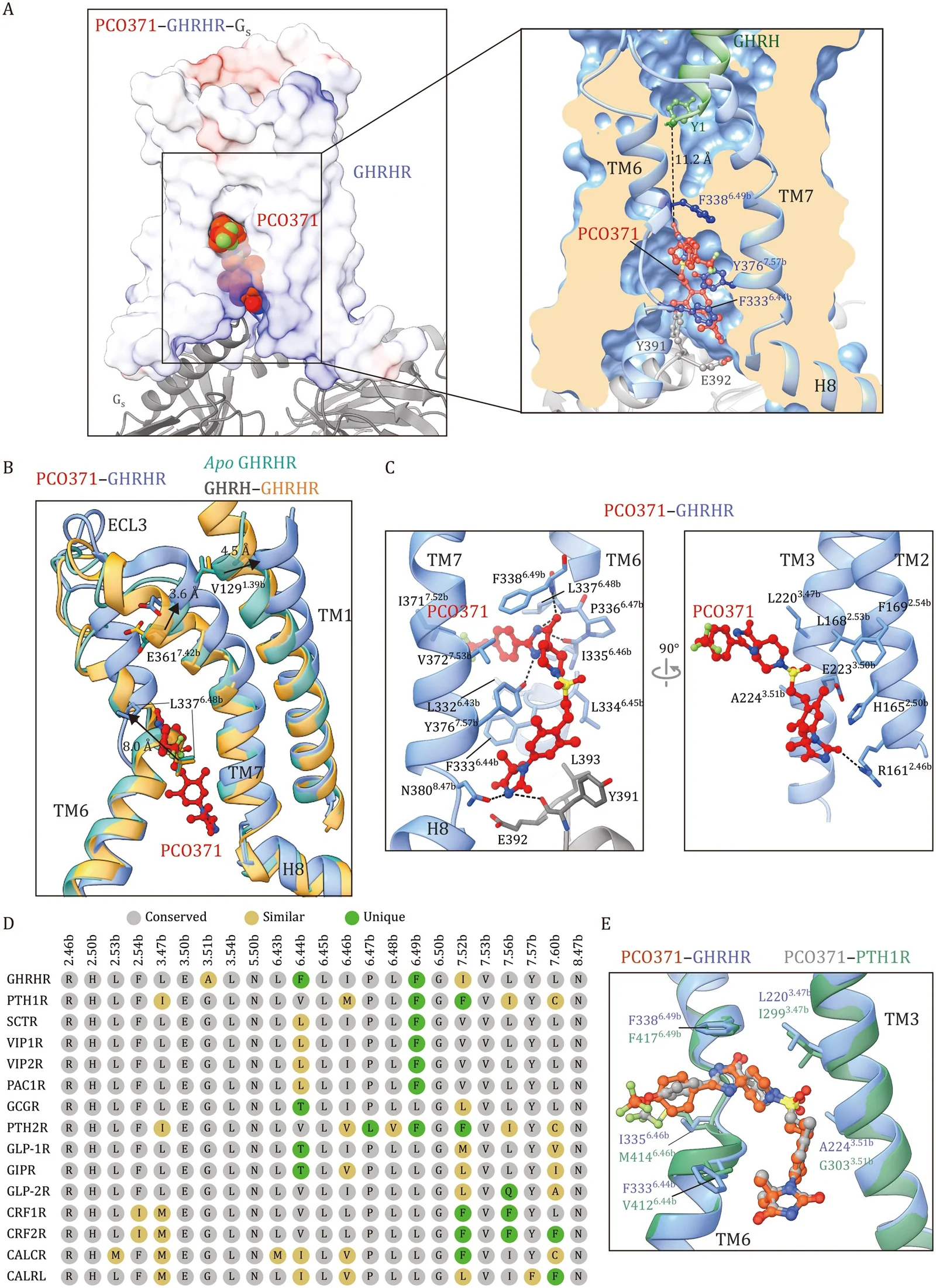
Allosteric binding of PCO371 to GHRHR. (A) Orthogonal views of the intracellular binding site of PCO371 in the PCO371–GHRHR–G_s_ complex (receptor shown as surface). The right panel provides a magnified view of the binding pocket. Architecturally, the pocket is constructed by the cytoplasmic segments of TM2, TM3, TM6 and TM7. Key gatekeeper residues are shown as blue sticks and labeled with the class B1 GPCR numbering in superscript (Wootten et al., 2013). PCO371 binds at an allosteric site distinct from the orthosteric pocket (occupied by GHRH, shown in cartoon) and is in close proximity to the C-terminal tip of the Gα_s_ α5-helix. (B) Structural comparison of *Apo*, GHRH-bound, and PCO371-bound GHRHR reveals that allosteric binding of PCO371 induces conformational rearrangements in TMs 1, 6, and 7. The Cα atom of E361^7.42b^ shifts by 3.6 Å, and the Cα of V129^1.39b^ moves by 4.5 Å when comparing the PCO371-bound state to the *Apo* state. (C) Close-up views of specific interactions between PCO371 and TM6/TM7 (left) or TM2/TM3 (right). Polar contacts are depicted as dashed lines. A critical structural feature is the direct interaction between PCO371 and the G protein, in which the base of PCO371 directly contacts the α5 helix of the Gα_s_ subunit. (D) Sequence alignment of key residues constituting the PCO371 allosteric pocket across human class B1 GPCRs. Within a 6 Å radius of the ligand, 20 residue positions are identical/similar between GHRHR and PTH1R, which explains the broad, albeit variable, activity of PCO371 across these receptors. (E) Structural superposition of PCO371 bound to PTH1R and GHRHR. Residues that differ between the two receptors at the binding interface are highlighted in stick representation.

Of note, PCO371 binding elicits a distinct and localized conformational adjustment within the TM6 (Figs. 2B and S5). To avoid severe steric clash with PCO371, the TM6 kink region undergoes a marked outward displacement and partial unwinding. Quantitatively, the Cα atom of L337^6.48b^ in PCO371-bound GHRHR shifts by 7.95 Å relative to its position in the *Apo* state and by 8.46 Å relative to the GHRH-bound state (Fig. 2B). Such a local perturbation propagates to the extracellular side of the receptor, leading to notable displacements in ECL3 and the extracellular tips of TM7 and TM1 (Fig. 2B). In contrast, TMs 2–5 exhibit highly aligned structural features across *Apo*, GHRH-bound, and PCO371-bound complexes (Fig. S5). This specific pattern indicates that the allosteric modulation of PCO371 is remarkably precise, primarily perturbing the helices (TM1, TM6, and TM7) that directly construct its binding site and associated extracellular loops, while leaving the structural framework of the active receptor largely unchanged.

PCO371 adopts a U-shaped conformation, stabilized by extensive interactions with both GHRHR and Gα_s_ (Figs. 2C and S6). The dimethylhydantoin group, positioned deeply in the pocket, acts as a critical anchor to the G protein interface by forming a salt bridge with R161^2.46b^. Furthermore, it engages in polar interactions with the backbone atoms of Y391 (Gα_s_) and the sidechain atom of N380^8.47b^ (GHRHR). The central dimethylphenyl group is stabilized by robust aromatic stacking interactions with H165^2.50b^, Y376^7.57b^, and Y391 (Gα_s_). The methyl substituents on this phenyl ring project into hydrophobic sub-pockets formed by L227^3.54b^, L334^6.44b^, and L394 (Gα_s_), effectively locking this group in place. The spiro-imidazolone moiety is advantageously lodged within the remodeled kink of TM6, where it forms multiple hydrogen bonds with Y376^7.57b^ and the mainchain atoms of three TM6 residues (I335^6.46b^, L337^6.48b^, and F338^6.49b^). These polar interactions act as a molecular staple, rigidly anchoring the ligand to TM6, thereby accounting for the necessity of the local helical rearrangement (Fig. 2C). Finally, the trifluoromethoxyphenyl tail extends to the narrow cleft between TM6 and TM7, where its phenyl ring engages in stacking interactions with F338^6.49b^ and is simultaneously enveloped by a hydrophobic sleeve formed by L332^6.43b^, I335^6.46b^, L337^6.48b^, I371^7.52b^, and L375^7.56b^, while its trifluoromethoxy group projects toward the lipid-facing exterior (Fig. S6A).

Although sequence alignment reveals that the PCO371-binding pocket is highly conserved across class B1 GPCRs (Kobayashi et al., 2023; Zhao et al., 2023), a notable distinction lies within TM6 (Fig. 2D). In GHRHR, two consecutive positions (6.44 b and 6.49 b) are occupied by large aromatic phenylalanine residues (F333^6.44b^ and F338^6.49b^), creating a unique, continuous aromatic surface on one side of the binding pocket. In contrast, PTH1R features a valine at position 6.44 b and a phenylalanine only at 6.49 b, and other class B1 receptors possess smaller aliphatic residues (e.g., leucine, valine, or threonine) at one or both of these positions. This dual-phenylalanine motif in GHRHR provides a more extensive and complementary aromatic environment for the central dimethylphenyl and tail phenyl groups of PCO371 (Figs. 2E and S6D). The enhanced van der Waals contacts and optimized π-π stacking interactions afforded by this configuration are likely to enhance binding affinity and more effectively stabilize the active conformation of the receptor–G protein complex (Fig. S7), thus constituting a key structural basis for the observed superior agonist potency of PCO371 at GHRHR compared to PTH1R (Kobayashi et al., 2023). This analysis highlights how subtle variations within a conserved allosteric pocket, i.e., single-residue differences, can be exploited to achieve receptor subtype selectivity, offering a precise template for structure-based design of next-generation, selective allosteric modulators for class B1 GPCRs.

To fully explore the conformational spectrum of GHRHR, we undertook a parallel effort to resolve its inactive, antagonist-bound structure in the absence of intracellular effectors (Fig. S8). Negative-staining and initial cryo-EM analyses demonstrated sample homogeneity and the presence of the complex (Fig. S8D and S8E). However, 2D class averages indicated incomplete Fab occupancy and potential flexibility at fusion junctions, thereby precluding high-resolution 3D reconstruction from the current dataset (Fig. S8F). Given the challenges in obtaining a high-resolution structure of the antagonist-bound GHRHR via cryo-EM, we turned to MD simulations (Figs. S9 and S10). MIA-602 functions as an antagonist by enforcing a specific inactive conformational ensemble of GHRHR through a multi-tiered mechanism. Its focused binding mode restricts receptor dynamics and prevents the compaction of the central polar network, thereby aborting signal initiation (Fig. S10A-D). Consequently, the conformational coupling between TM6 and TM7 via the H165^2.50b^–E223^3.50b^–T331^6.42b^–Y376^7.57b^ (HETY) motif is halted, and the intracellular domain is maintained in a dilated, G protein-incompatible state due to the disassembly of the TM2–TM6–TM7–H8 polar network (Zhang et al., 2017) (Fig. S10E and S10F). This hierarchical suppression of the activation steps explains the potent and sustained antagonism exerted by MIA-602.

By integrating cryo-EM and MD simulations, this study delineates the structural landscape of the human GHRHR across key conformational states: the ligand-free (*Apo*) G_s_-coupled state, the allosteric small-molecule agonist (PCO371)-bound G_s_-coupled state, and the peptidic antagonist (MIA-602)-bound inactive state (without G protein coupling). The endogenous ligand GHRH triggers large-scale inward folding of ECL1, which acts as an extracellular clamp essential for full activation. The allosteric agonist PCO371 remodels a conserved intracellular pocket, functioning as an intracellular wedge that stabilizes the active G_s_ interface without inducing extracellular reorganization, thereby establishing a structural basis for biased signaling. Conversely, the antagonist MIA-602 enforces an inactive state by locking the conserved intracellular HETY motif, preventing the characteristic outward swing of TM6 required for G protein engagement. Our findings collectively underscore that GHRHR signaling preference, namely, its propensity to engage specific transducers in response to different ligand stimulation, is governed by distinct, ligand-specific conformational adaptations at both extracellular and intracellular interfaces (Supplementary Materials). By linking atomic-level structural adaptations to functional signaling preferences, this work offers valuable insights into the development of next-generation GHRHR therapeutics.

## Supplementary Material

pwag016_Supplementary_Data

## Footnotes

We are grateful to Shi Lin for technical assistance. The cryo-EM data were collected at Cryo-Electron Microscopy Research Center and Advanced Center for Electron Microscopy, Shanghai Institute of Materia Medica, Chinese Academy of Sciences.

This work was partially supported by the National Natural Science Foundation of China (32200576 to Z.T.C., 82273961, 82073904, and 81872915 to M.-W.W.), The Explorers Program of Shanghai (Basic Research Funding, 25TS1400700 to Q.T.Z.), STI2030-Major Project 2021ZD0203400 (to Q.T.Z.) and Hainan Provincial Major Science and Technology Project ZDKJ2021028 (to Q.T.Z.).

The authors declare no conflict of interest. The authors declare their agreement to publish. This paper does not report original code.

All relevant data are available from the authors and/or included in the manuscript or Supplementary Information. The cryo-EM density maps have been deposited in the Electron Microscopy Data Bank (EMDB) under accession codes EMD-69803 (*Apo* GHRHR–G_s_) and EMD-69804 (PCO371-bound GHRHR–G_s_). Coordinates have been deposited in the Protein Data Bank (PDB) under accession codes 24TB (*Apo* GHRHR–G_s_), and 24TI (PCO371-bound GHRHR–G_s_).

K.N.H., Y.Q., X.Y.C., Y.Y.C., S.T.H. and Q.T.Z. performed research; K.N.H., Y.Q., Q.T.Z. and M.-W.W. analyzed the data; Q.T.Z., K.N.H and M.-W.W. wrote the manuscript with input from all coauthors; M.-W.W. conceived the idea, initiated the project; M.-W.W., Q.T.Z. and Z.T.C. supervised the studies.
