# Supplementary material for: Structural adaptation associated with signaling preference at the human Growth Hormone–Releasing Hormone Receptor
Source: Protein Cell. 2026 Mar 18;17(9):883–8. doi: 10.1093/procel/pwag016 (PMC13521273; doi:10.1093/procel/pwag016)
Supplement: Supplementary file 1 [file pwag016_supplementary_data.pdf]

Supplemental Information

Structural adaptation associated with signaling preference at the human GHRHR

Ming-Wei Wang[1,2,3,4,5,*,†], Kaini Hang[1,†], Yue Qiu[6,†], Xianyue Chen[2], Yanyan Chen[2], Shengtian Huang[1], Zhaotong Cong[1,7], Qingtong Zhou[3,*]

[1]Department of Pharmacology, School of Basic Medical Sciences, Fudan University, Shanghai 200032, China;

[2]Department of Biology, Research Center for Deepsea Bioresources, Sanya 572025, China;

[3]Research Center for Medicinal Structural Biology, National Research Center for Translational Medicine at Shanghai, State Key Laboratory of Medical Genomics, Ruijin Hospital, Shanghai Jiao Tong University School of Medicine, Shanghai 200025, China;

[4]Engineering Research Center of Tropical Medicine Innovation and Transformation of Ministry of Education, School of Pharmacy, Hainan Medical University, Haikou 570228, China;

[5]Department of Chemistry, School of Science, The University of Tokyo, Tokyo 113-0033, Japan;

[6]Jinhua Key laboratory of Quality Evaluation and Standard Research of Traditional Chinese Medicine, Jinhua Institute for Food and Drug Control, Jinhua Advanced Research Institute of Technology, Jinhua 321019, China;

[7]Institute of Herbgenomics, Chengdu University of Traditional Chinese Medicine, Chengdu 611137, China.

[†]These authors contributed equally to this work.

[*]Correspondence: mwwang@simm.ac.cn (M.-W.W.) or zhouqt@sjtu.edu.cn (Q.T.Z.).

Materials and methods

Cell culture

Spodoptera frugiperda (*Sf*9) insect cells were grown in ESF 921 serum-free medium (Expression Systems, Davis, CA, USA) at 27°C and 120 r.p.m. (Infors HT, Bottmingen, Switzerland). HEK293T cells were purchased from the Cell Bank at the Chinese Academy of Sciences, cultured in Dulbecco's modified Eagle's medium (DMEM; Life Technologies, Carlsbad, CA, USA) supplemented with 10% fetal bovine serum (FBS; Gibco, Carlsbad, CA, USA) at 37°C in 5% CO_2 humidified incubator (ThermoFisher Scientific, Waltham, MA, USA).

Constructs

For structural studies, wild-type (WT) human GHRHR DNA (Genewiz/Azenta, Indianapolis, IN, USA) was cloned into a pFastBac vector (Invitrogen, Carlsbad, CA, USA), where its native signal sequence (M1–G22) was replaced by a hemagglutinin (HA) signal peptide. The construct was further modified through sequential C-terminal truncation of residues A406–C423, fusion to the LgBiT subunit (Promega, Madison, WI, USA) via a 15-amino acid polypeptide (GSSGGGGSGGGGSSG) linker to facilitate the NanoBiT tethering strategy (Duan et al., 2020), insertion of a TEV protease cleavage site, and addition of a tandem maltose-binding protein (MBP) tag to facilitate expression and purification.

The G_s chimeric construct (G112) incorporated a mini-G_s (Zhou et al., 2021) structural scaffold originally developed for resolving the A_2AR–mini-G_s crystal structure (Carpenter et al., 2016). Rat Gβ1 with an N-terminal 8× His-tag was fused with a SmBiT (Dixon et al., 2016) (peptide 86, Promega) by a 15-amino-acid polypeptide linker at the C-terminus. G112, rat Gβ1, and bovine Gγ2 were cloned into pFastBac vectors individually.

Nanobody 35

Nanobody 35 (Nb35) with a C-terminal 6× His-tag was expressed in the periplasm of *Escherichia coli (E. coli)* BL21 (DE3) cells and cultured in Terrific Broth (TB) medium supplemented with 100 μg mL^{-1} ampicillin, 2 mM $MgCl_2$, 0.1% glucose at 37°C for 2.5 h until an optical density of 0.7–1.2 at 600 nm (OD_{600}) was reached. Protein expression was induced with 1 mM isopropyl β-D-1-thiogalactopyranoside (IPTG) overnight at 18°C. Then the cells were collected and disrupted by ultrasonication in lysis buffer (20 mM Tris-HCl, pH 8.0, 300 mM NaCl and 20 mM imidazole), and the Nb35 protein was purified by Ni-NTA affinity chromatography, followed by size-exclusion chromatography (SEC) on a Superdex™ 200 Increase 10/300 GL column (Cytiva, Marlborough, MA,

USA) equilibrated with gel filtration buffer (20 mM HEPES, pH 7.4, 150 mM NaCl). Peak fractions were pooled and verified by SDS–polyacrylamide gel electrophoresis. Purified Nb35 was concentrated to 5–10 mg/mL, flash-frozen in liquid nitrogen, and stored at -80°C.

Expression and purification

GHRHR–15AA–LgBiT–2MBP, G112, His10–Gβ1–peptide 86, and Gγ2 recombinant baculoviruses were individually prepared using the Bac-to-Bac Baculovirus Expression System (Invitrogen). The *Sf*9 insect cells were cultured to a density of 3×10^6 cells per mL in ESF 921 cell culture medium (Expression Systems) and then coinfected with four distinct viruses at a ratio of 1:3:3:3 corresponding to GHRHR, G112, Gβ1, and Gγ2. Cells were harvested by centrifugation 48 h post infection and washed once with PBS, then the cell pellets were stored at -80°C until use.

The cell pellets were thawed on ice and lysed in a lysis buffer composed of 20 mM HEPES, pH 7.5, 100 mM NaCl, 10% (v/v) glycerol, 10 mM $MgCl_2$, 5 mM $CaCl_2$, 1 mM $MnCl_2$, and 100 μM TCEP, with the addition of EDTA-free protease inhibitor cocktail (Bimake, Houston, TX, USA) by dounce homogenization. To initiate the formation of *Apo* state GHRHR–G_s complex, 10 μg/mL Nb35, 25 mU/mL apyrase (Sigma-Aldrich, St. Louis, MO, USA) was added to the cell lysate and the mixture was incubated for 1.5 h at room temperature (RT). For the PCO371–GHRHR–G_s complex, 200 μM PCO371 was additionally added to the mixture. The membrane was further solubilized by 0.5% (w/v) lauryl maltose neopentyl glycol (LMNG; Anatrace) and 0.1% (w/v) cholesterol hemisuccinate (CHS; Anatrace) for 2 h at 4°C. After centrifugation at 65,000× g for 30 min, the supernatant was isolated and incubated with amylose resin (NEB) for 2 h at 4°C. The resin was then collected by centrifugation at 600× g for 10 min and loaded into a gravity flow column (Sangon Biotech, Shanghai, China). The column was first washed with five column volumes of buffer containing 20 mM HEPES, pH 7.5, 100 mM NaCl, 10% (v/v) glycerol, 5 mM $MgCl_2$, 1 mM $MnCl_2$, 25 μM TCEP, 0.1% (w/v) LMNG, and 0.02% (w/v) CHS, followed by washing with 15 column volumes of buffer containing 20 mM HEPES, pH 7.5, 100 mM NaCl, 10% (v/v) glycerol, 5 mM $MgCl_2$, 1 mM $MnCl_2$, 25 μM TCEP, 0.03% (w/v) LMNG, 0.01% (w/v) glyco-diosgenin (Anatrace), and 0.008% (w/v) CHS. The protein was subsequently incubated overnight at 4°C with His-tagged TEV protease (customer-made) on the column to remove the C-terminal 2MBP-tag in the buffer above. The flow through was collected next day and concentrated with a 100 kDa molecular weight cut-off (MWCO) concentrator (Millipore/Merck KGaA, Darmstadt, Germany). Concentrated GHRHR–G_s complex or PCO371–GHRHR–G_s complex was loaded onto a Superdex 200 increase 10/300 GL column (GE Healthcare,

Buckinghamshire, UK) with running buffer containing 20 mM HEPES, pH 7.5, 100 mM NaCl, 10 mM MgCl2, 100 μM TCEP, 5 μM GHRH, and 0.001% digitonin (Anatrace, Maumee, OH, USA). The fractions for monomeric complex were collected and concentrated to 10–20 mg/mL for cryo-EM examination.

Cryo-EM data acquisition

The purified *Apo* GHRHR–G_s and PCO371–GHRHR–G_s complexes at a concentration of 15–20 mg/mL were applied onto glow-discharged Quantifoil holey carbon grids (R1.2/1.3, Au, 300 mesh), blotted using a Vitrobot Mark IV (ThermoFisher Scientific, Waltham, MA, USA) at 4°C in 100% humidity, and subsequently plunge-frozen in liquid ethane. The prepared grids were transferred onto a Titan Krios G4 (FEI) equipped with a K3 Summit direct electron detector (Gatan, Pleasanton, CA, USA) and cryo-EM images were automatically collected using Serial EM software (v.3.7.4)(Mastronarde, 2005) at a nominal magnification of 105,000 in counting mode, corresponding to a calibrated pixel size of 0.5355 Å per pixel at the Cryo-Electron Microscopy Research Center at Shanghai Institute of Materia Medica, Chinese Academy of Sciences. In total, 6,324 movies for the *Apo* GHRHR–G_s complex and 8,071 movies for the PCO371–GHRHR–G_s complex were recorded at an accumulated total dose of 80 electrons per $Å^2$ for 36 frames, with a defocus range of –1.2 to –2.2 μm.

Image processing

All dose-fractionated image stacks were subjected to beam-induced motion correction and dose-weighting using MotionCor2.1 (Zheng et al., 2017). A sum of all frames, filtered according to the exposure dose, in each image stack was used for further processing. Contrast transfer function parameters for each micrograph were determined by Gctf v1.06. Further data processing was performed in cryoSPARC (Punjani et al., 2017). Particle selection, two-dimensional classification, and the first round of three-dimensional classification were performed on a binned dataset with a pixel size of 0.5355 Å. Auto-picking yielded 7,176,212 particle projections for *Apo* GHRHR–G_s complex and 4,122,670 particle projections PCO371–GHRHR–G_s complex that were subjected to reference-free two-dimensional classification to discard false-positive particles or particles categorized in poorly defined classes for further processing. This subset of particle projections was subjected to consecutive rounds of three-dimensional classification with a pixel size of 0.5355 Å. Further three-dimensional classifications focusing on the complex produced one good subset with higher resolution, which was subsequently subjected to three-dimensional refinement and Bayesian

polishing. The final refinement generated a map with an indicated global resolution of 3.04 Å and 2.88 Å, respectively.

Model building and refinement

The GHRH–GHRHR–G_s complex structure (PDB ID: 7CZ5) was used as an initial template for model building. Lipid coordinates and geometry restraints were generated using phenix.elbow. Models were docked into the EM density map using UCSF Chimera. This starting model was then subjected to iterative rounds of manual adjustment and automated refinement in Coot v0.9.2(Emsley and Cowtan, 2004) and Phenix v1.19.1 (Adams et al., 2010), respectively. The final refinement statistics were validated using the module "comprehensive validation (cryo-EM)" in PHENIX. Structural figures were prepared in Chimera (Pettersen et al., 2004), Chimera X (Pettersen et al., 2021), and PyMOL (https://pymol.org/2/). To facilitate the comparison of conserved residues across receptors, receptor residues were labeled in superscript according to the class B1 GPCR numbering scheme, wherein the first digit denotes the transmembrane helix, and the second number indicates the position relative to the most conserved residue (*e.g.*, $G^{6.50}$ corresponds to transmembrane helix 6, where .50 is the most conserved residue)(Wootten et al., 2013).

MD simulation

The initial structural model of the antagonist MIA-602-bound GHRHR was constructed using the inactive GHRHR structure (https://gpcrdb.org/structure/homology_models/ghrhr_human_inactive, predicted by AlphaFold2-Multistate (Pandy-Szekeres et al., 2023)) as a template via progressive mutation of the aligned GHRH structure. The model was then refined using the Protein Preparation Wizard (Schrödinger, v2023-2), involving addition of missing atoms/loops, assignment of protonation states at pH 7.0, and energy minimization. Noncanonical residues in MIA-602 (*e.g.*, pentafluorophenyl-Phe, Orn, homo-Arg, O-Me-Tyr, and norleucine) were represented by their CHARMM residue types; D-amino acids used standard D-variants. Owing to the significant complexity in predicting the conformation of the long N-terminal PhAc-Ada lipid modification and the fact that analogous compounds lacking this group maintain similar inhibitory efficacy, the precise PhAc-Ada conformation was not modeled. Instead, the N-terminus was capped with an acetyl group.

Systems were built with CHARMM-GUI Membrane Builder (Lee et al., 2016), embedded in a hydrated lipid bilayer, and solvated with explicit TIP3P water at 0.15 M NaCl. The CHARMM36m force field (Huang et al., 2017) was used for protein (CHARMM36 for lipids), long-range electrostatics were treated by PME with a 1.2 nm real-space cutoff, and Lennard-Jones interactions

utilized a force-switch from 1.0 to 1.2 nm; bonds to hydrogens were constrained with LINCS under the Verlet cutoff scheme. After steepest-descent minimization, six NPT equilibration stages with gradually reduced positional/dihedral restraints were performed (dt = 1 fs for stages 1–3 and 2 fs for stages 4–6; 2.5 ns and 5.0 ns per stage, respectively; total 22.5 ns) using a Berendsen thermostat (τT = 1.0 ps; coupling groups PROT/MEMB/SOL_ION) and a semi-isotropic Berendsen barostat (τP = 5.0→2.0 ps). Finally, restraint-free production MD was run in the NPT ensemble at 310 K and 1 bar with a 2 fs time step, a V-rescale thermostat (τT = 1.0 ps) and a semi-isotropic Parrinello–Rahman barostat (τP = 2.0 ps), yielding 1 μs trajectories per system. System stability was verified by monitoring backbone root-mean-square deviation (RMSD), temperature, pressure, and total energy over time.

All trajectory analyses were conducted using the built-in modules of Gromacs. The 1 μs production trajectories were preprocessed through a standard post-treatment workflow to remove artifacts arising from periodic boundary conditions and overall system rotation and translation. RMSD, root-mean-square fluctuation (RMSF), and radius of gyration (Rg) were computed to evaluate the complex structural stability. The inactive GHRHR+MIA-602 contact surface area was quantified based on solvent-accessible surface area (SASA) calculations. In addition, residue-resolved contact persistence scoring (RRCS) was performed using the standalone gmx_RRCS package (Han et al., 2025, Zhou et al., 2019) to quantify per-residue contact contributions along the MD trajectory. Briefly, residue–residue contact events were calculated frame-by-frame using a distance-based cutoff (3.23–4.63 Å), and the resulting contact time-series was converted to RRCS values as defined in the original method (Han et al., 2025). For each residue, the mean RRCS value over the last 500 ns of the simulation (500-1000 ns) was used (i) as the quantitative metric reported in this study and (ii) to generate the ranked RRCS bar plots.

Supplementary results

Structure determination

In order to solve the cryo-EM structures of *Apo* GHRHR–G_s and PCO371–GHRHR–G_s complexes, we applied the previously characterized construct of GHRHR (Zhou et al., 2020) and utilized the NanoBiT tethering strategy (Zhao et al., 2021) to stabilize the assembly of complexes. The N-terminal native signal peptide of GHRHR was replaced by a hemagglutinin (HA) signal peptide and the carboxy terminus of GHRHR was truncated to residue 405 to increase its expression level. These previously validated modifications do not affect ligand binding or receptor activation (Zhou et al., 2020). The complexes were purified by SEC and verified by SDS gel electrophoresis (**Figs. S1A** and **S2A**). The extracellular domain (ECD) of GHRHR was invisible in both structures owing to the flexibility of the ECD in the absence of orthosteric peptide binding (**Figs. S1** and **S2**).

GHRHR in *Apo* state

The conformation of the *Apo* GHRHR is also closely aligned with the active, G_s-coupled states of GHRH-bound SV1 (PDB: 7V9M; Cα RMSD = 0.96 Å) and *Apo* SV1 (PDB: 7V9L; Cα RMSD = 0.93 Å) (**Fig. S4B**). In contrast, the *Apo* GHRHR–G_s complex diverges significantly from the canonical inactive conformation of class B1 GPCRs, as exemplified by the PF-06372222-bound inactive GLP-1R (PDB: 5VEW)(Song et al., 2017) (Cα RMSD = 1.70 Å) (**Fig. S4C**). The inward movements of TM6 thereby open the intracellular crevice for G protein coupling, which is a hallmark of receptor activation. Notably, TM6 is well-aligned across all four determined G_s-coupled GHRHR structures (*Apo* WT, GHRH-bound WT, GHRH-bound SV1, and *Apo* SV1), indicating that this conserved architecture is an essential feature for G protein coupling (**Fig. S4A and S4B**). Collectively, these results indicate that the ligand-free, G_s-coupled GHRHR displays an active-like state.

PCO371-bound GHRHR

Comparison of PCO371-bound state with that of *Apo* and GHRH-bound structures shows that PCO371 stabilizes a canonical active conformation of the receptor, characterized by the hallmark outward movement of the intracellular end of TM6 and a complementary inward shift of TM5, opening the cytoplasmic crevice for G_s engagement, a signature shared with the GHRH-bound state (**Fig. S5**). While the intracellular ends of TM6 and TM7 maintain a conserved architecture across all three states, a pronounced rearrangement occurs at the conserved proline-induced kink in the middle of TM6 (**Fig. S5**).

G protein coupling

Similar to other class B1 GPCRs, GHRHR couples to the G_s protein through a canonical intracellular cavity formed by the outward movement of TM6 and the concerted shifts of TM5 and TM7 upon activation (**Fig. S7**). The α5-helix of $Gα_s$ inserts into this crevice, engaging a conserved set of residues from TM2, TM3, TM5, TM6, TM7, H8 and ICL2. Such a core interaction architecture is maintained across the *Apo*, PCO371-bound, and GHRH-bound GHRHR–G_s complexes, facilitating G protein engagement in all three states (**Fig. S7A**). Despite this conserved interface, detailed structural alignment reveals a ligand-dependent, torsional shift of the entire G_s heterotrimer relative to the receptor core (**Fig. S7B**). It is most pronounced in the αN-helix and Ras-like domain of $Gα_s$. Relative to the fully active, GHRH-bound state, the αN-helix rotates outward, with a displacement of approximately 4.6 Å at the Cα of E15 in the PCO371-bound and 8.0 Å in the *Apo* complexes. Concomitant movements are observed in the Ras-like domain (α4 helix), shifting by 1.8 Å (Cα of T350) in the PCO371-bound state and 1.2 Å in the *Apo* state. This stepwise outward movement of G_s perturbs the receptor–G protein interface in a state-specific manner. For instance, Q381 in the *Apo* and PCO371-bound structures rotates outward, preventing it from forming the hydrogen bond with E392 observed in the GHRH-bound state. The structural gradation—from the fully engaged GHRH-bound state to the partially displaced PCO371-bound state and the fully displaced *Apo* state—directly mirrors the functional hierarchy: full agonism (cAMP accumulation) by GHRH, partial agonism by PCO371, and basal activity in the *Apo* state. The absence of the stabilizing nanobody Nb35 in the *Apo* structure further suggests inherent instability or an allosterically altered conformation of the G_s heterotrimer in the basal state (**Fig. 1A**). Smaller, concerted displacements are also observed in the Gβ and Gγ subunits.

Antagonist-bound GHRHR

Due to lack of a generalizable strategy for determining inactive class B1 GPCR structures by cryo-EM, we engineered a chimeric receptor by inserting a BRIL domain into ICL3 (**Fig. S8**). This approach aimed to form a stable complex with an anti-BRIL Fab and an anti-Fab nanobody, providing a distinct structural feature for particle alignment, a strategy successfully adapted from work on class A GPCRs (Zhang et al., 2022, Chen et al., 2022, Guo et al., 2024). We utilized AlphaFold3 to screen over two hundred potential BRIL insertion sites, prioritizing constructs predicted to form continuous helical connections between BRIL and the receptor. Experimental validation identified the construct with BRIL inserted between residues 309 and 321 as optimal, based on its high expression yield and clear appearance in SEC (**Figs. S8A and S8B**). This construct successfully formed a ternary complex

with the Fab and nanobody, as confirmed by SEC and SDS-PAGE analysis (**Fig. S8C**). These negative-staining and cryo-EM analyses results show an overall complex assembly and highlight the need for further construct or sample optimization to achieve a stable, rigid complex suitable for atomic-resolution structure determination (**Fig. S8D-F**). Therefore, we adopted MD simulations to investigate the conformational dynamics of antagonist-bound GHRHR relative to its active state.

The MD simulations system was constructed based on an inactive GHRHR model predicted by AlphaFold2-Multistate (Pandy-Szekeres et al., 2023), embedded in a lipid bilayer, solvated with explicit TIP3P water, and subjected to all-atom MD simulations. Across four independent 1 μs replicate simulations, the MIA-602–GHRHR complex demonstrated consistent structural stability (**Figs. S9 and S10**). The backbone RMSD plateaued after initial equilibration, ligand RMSD remained low, the transmembrane core exhibited reproducible rigidity, and radius of gyration was sustained (**Fig. S9A-C**). These stable trajectories provide a reliable basis for residue-level comparison with active state structures to elucidate the atomic mechanism of GHRHR antagonism. A hallmark of receptor activation is the conformation of the intracellular end of TM6, which swings outward in the active state to accommodate G protein coupling but remains inward and packed against TM3 and TM7 in the inactive state (Cong et al., 2021a, Zhang et al., 2017). As shown in **Fig. S10C**, throughout the simulations of antagonist-bound GHRHR, the intracellular end of TM6 consistently maintained an inward, inactive conformation, distinctly different from the outward displacement characteristic of the active GHRHR. This sustained inactive TM6 geometry indicates that MIA-602 binding effectively prevents the conformational change required for G protein engagement.

When looking at the peptide-binding modes, fundamental differences between MIA-602 and GHRH bound (PDB: 7CZ5) structures were found, correlating with their opposing functionalities, antagonism *vs*. agonism. MIA-602 was compact and highly conserved across the MD simulations, stabilized by a focused, persistent core of receptor residues (*e.g.*, E361, F105, H341 and D350) (**Fig. S10B**). Quantitative residue-residue contact score (RRCS) analysis confirmed that antagonist–GHRHR contact is dominated by a small subset of residues, with a sharp decline in contribution beyond this core cluster, indicative of a focused interaction epitope (**Fig. S9D**). In contrast, GHRH in active state adopted an extended binding mode, engaging a broader set of receptor residues (*e.g.*, K182, H210, A271 and D274) along the binding crevice. This suggests that GHRHR agonism requires a multi-point, distributed interaction network to induce specific conformational changes, whereas antagonism by MIA-602 is achieved through a focused, rigid binding mode that restricts receptor

dynamics.

Following the central polar network at the base of the orthosteric pocket responsible for signal initiation, class B1 GPCR activation involves sequential reorganization of two additional polar networks (Cong et al., 2021a, Zhang et al., 2017). These include the rearrangement of the conserved HETY motif (H165$^{2.50b}$–E223$^{3.50b}$–T331$^{6.42b}$–Y376$^{7.57b}$) critical for signal propagation, and the intracellular polar network connecting TM2, TM6, TM7 and H8 vital for stabilizing the G protein coupling interface. We quantified the geometry of these networks by comparing minimum inter-residue distances in the active-state cryo-EM structure with those from our MD trajectories of the MIA-602-bound complex (**Fig. S10D-F**). For conserved HETY motif, the distance between H165$^{2.50b}$ and T331$^{6.42b}$ averaged 5.8–7.0 Å, significantly shorter than 14.3 Å observed in the active state. Similarly, the T331$^{6.42b}$–Y376$^{7.57b}$ distance remained short (mean value ~3.6 Å) compared to the corresponding distance (8.3 Å) in the GHRH–GHRHR complex (**Fig. S10E**). Although transient movements were sampled, the HETY motif was stably coupled, preventing propagation of activation signal. For the TM2–TM6–TM7–H8 polar network, notable structural fluctuations and dynamics were observed, but the conformation remained inactive-like and distant from the fully active state structure. For example, the minimum distances between the sidechains of R161$^{2.46b}$ and T331$^{6.42b}$ exhibited mean values of 3.4–6.2 Å during the MD simulation, whereas in the active state structure this distance is 15.9 Å (**Fig. S10E**). A similar phenomenon was found for the minimum distances between the sidechains of R326$^{6.37b}$ and Q381$^{8.48b}$, where two representative conformations were observed but both were distinct from the fully active state (**Fig. S10F**). This sustained, closed intracellular architecture is unfavorable for G protein binding.

Supplementary discussion

GHRHR is a central endocrine GPCR governing systemic growth and metabolism, whose dysregulation leads to growth disorders, while aberrant signaling, particularly through oncogenic splice variants like SV1, is implicated in certain cancers (Granata et al., 2025, Gesmundo et al., 2025). This duality establishes GHRHR as a compelling and clinically validated drug target. The approval of the GHRHR agonist tesamorelin for HIV-associated lipodystrophy validates the therapeutic potential of targeting this pathway (Stanley et al., 2014). Concurrently, antagonists such as MIA-602 show promise in pre-clinical models of hormone-dependent tumors (Perez et al., 2012, Fahrenholtz et al., 2014). The emergence of diverse ligand chemotypes, from peptide analogs tesamorelin and MIA-602 to orally bioavailable small molecules like PCO371, has expanded pharmacological tools

and therapeutic candidates. It also underscores the pressing need to mechanistically understand how occupancy of different topographical sites (orthosteric *vs.* allosteric) translates to functional selectivity (*e.g.*, G_s bias *vs.* antagonism)(Tamura et al., 2016, Zhao et al., 2023, Kobayashi et al., 2023). The three new structural states reported here (two cryo-EM structures, *Apo* GHRHR–G_s and PCO371–GHRHR–G_s, and one MD-simulated antagonist MIA-602–GHRHR complex), combined with our three previously published structures (GHRH-bound GHRHR–G_s, GHRH-bound SV1–G_s and *Apo* SV1–G_s)(Cong et al., 2021b, Zhou et al., 2020), provide a comprehensive atlas of the distinct GHRHR functional states. This rich dataset enables a detailed dissection of receptor architecture adaptation upon ligand binding to dictate signaling output.

A key finding relates to the remarkable structural divergence in the extracellular regions of GHRHR across its active states, contrasted with a highly conserved intracellular G_s-coupling architecture where the cytoplasmic ends of TMs 2-6 align nearly perfectly in the *Apo*, GHRH-bound, and PCO371-bound complexes. This extracellular plasticity is most pronounced in ECL1. Upon binding GHRH, ECL1 folds inward by over 16 Å to cradle the middle region of the peptide, forming a network of specific hydrogen bonds and stacking interactions that lock the agonist in place (Zhou et al., 2020). In the *Apo* state, ECL1 adopts a naturally extended and splayed conformation, stabilized by an intramolecular polar network, resulting in a wide-open extracellular pocket. This intrinsic flexibility of ECL1 highlights its crucial role as a conformational sensor and effector, suggesting that the kinetics and stability of ECL1 engagement could be a vital determinant for ligand efficacy and bias, and thus offers a novel design axis for engineering peptide agonists with tailored signaling profiles (Sun et al., 2020, Li et al., 2024, Cary et al., 2023). Furthermore, unlike the *Apo* G_s-coupled GCGR or GIPR, whose orthosteric pockets are partially occluded by inward-folded ECL2 (GCGR) or the TM6–ECL3 junction (GIPR)(Cong et al., 2024), the *Apo* GHRHR does not occlude its orthosteric site with inward-folded loops, maintaining an accessible pathway akin to that of GLP-1R (Cong et al., 2024). This feature may facilitate rapid peptide engagement and represents a unique functional adaptation within this receptor subclass.

The cryo-EM structure of the PCO371-bound GHRHR–G_s complex reveals an allosteric activation mechanism that bypasses extracellular reorganization. PCO371 occupies a well-defined intracellular pocket nestled between TM2, TM3, TM6 and TM7, more than 11 Å away from the orthosteric peptide-binding site. The U-shaped structure of PCO371 acts as a molecular wedge that stabilizes the pre-existing active-like conformation observed in the *Apo* state through highly localized

yet consequential conformational adjustment. To accommodate its bulky spiro-imidazolone group, the conserved kink in TM6 undergoes a pronounced outward displacement (~8 Å), a specific adaptation absent in the GHRH-bound state. This local perturbation propagates modestly to the extracellular tips of TM1 and TM7 but leaves the core scaffold (TMs 2-5) virtually unchanged. This demonstrates remarkable precision in allosteric modulation: PCO371 selectively remodels the helices that form its binding site to stabilize the active interface, particularly with the $G\alpha_s$ α5-helix which directly plugs the base of the pocket. Comparative analysis with PCO371-bound PTH1R confirms a conserved activation mechanism via this intracellular allosteric site (Zhao et al., 2023, Kobayashi et al., 2023). Of note, GHRHR possesses a unique dual-phenylalanine motif ($F333^{6.44b}$ and $F338^{6.49b}$) on TM6 that creates an extended aromatic surface, providing enhanced van der Waals and π-stacking interactions with PCO371. This subtle variation within a conserved pocket provides an explicit structural explanation as to the superior potency of PCO371 at GHRHR and offers a precise blueprint for designing GHRHR-selective allosteric agonists with optimized efficacy.

Determining high-resolution inactive state structures for class B1 GPCRs remains a significant technical hurdle. While strategies successful for class A GPCRs, such as BRIL insertion and antibody binding, have been adapted (Zhang et al., 2022, Chen et al., 2022, Guo et al., 2024), their application to the distinct transmembrane topology and dynamics of class B1 GPCRs, including GHRHR, has not been straightforward, as our own experimental efforts indicated (**Fig. S8**). To circumvent this, we applied MD simulations to obtain a dynamic view of antagonist-bound state. It was found that MIA-602 enforces antagonism by stabilizing and locking the conserved HETY motif ($H^{2.50b}$–$E^{3.50b}$–$T^{6.42b}$–$Y^{7.57b}$) in its inactive conformation (Yang et al., 2021). In the active state, the HETY motif is disrupted to allow TM6 outward swing. In the presence of MIA-602, this motif remains tightly coupled (*e.g.*, $H165^{2.50b}$–$T331^{6.42b}$ distance ~5.8–7.0 Å *vs.* 14.3 Å in the active state), acting as a primary "conformational lock" that prevents the propagation of the activation signal. Consequently, TM6 is held inward, which in turn maintains the intracellular polar network in a compact, closed configuration that is incompatible with G_s protein engagement. The focused binding mode of MIA-602, targeting a persistent hotspot residue cluster, contrasts with the well-distributed interactions of GHRH, explaining its role in restricting essential receptor dynamics required for activation. Although the flexible ECD was not resolved in the cryo-EM structures, our MD simulations allowed us to model this domain, revealing its likely role in mediating high-affinity binding of MIA-602. Future efforts combining engineered stabilizers for cryo-EM with advanced simulation methods will be

essential to capture a full atomic-resolution view of this inactive complex.

This study provides a unified structural framework for GHRHR, demonstrating how it is dynamically modulated by ligands through distinct extracellular or intracellular adaptations. The endogenous agonist GHRH acts as an extracellular clamp that stabilizes the active state by ordering the extracellular vestibule, particularly ECL1. The allosteric agonist PCO371 acts as an intracellular wedge, reinforcing the active state through local remodeling of its binding site. In contrast, the antagonist MIA-602 functions as a signaling blocker, stabilizing the inactive conformation by preventing the disruption of the HETY motif. This framework directly informs a new direction of relevant drug discovery: the elucidated flexibility of ECL1 presents a new region for peptide design; the detailed map of the PCO371 pocket enables structure-based design of selective non-peptide allosteric agonists; and the identification of the HETY motif as a key inhibitory node suggests novel strategies for antagonist development.

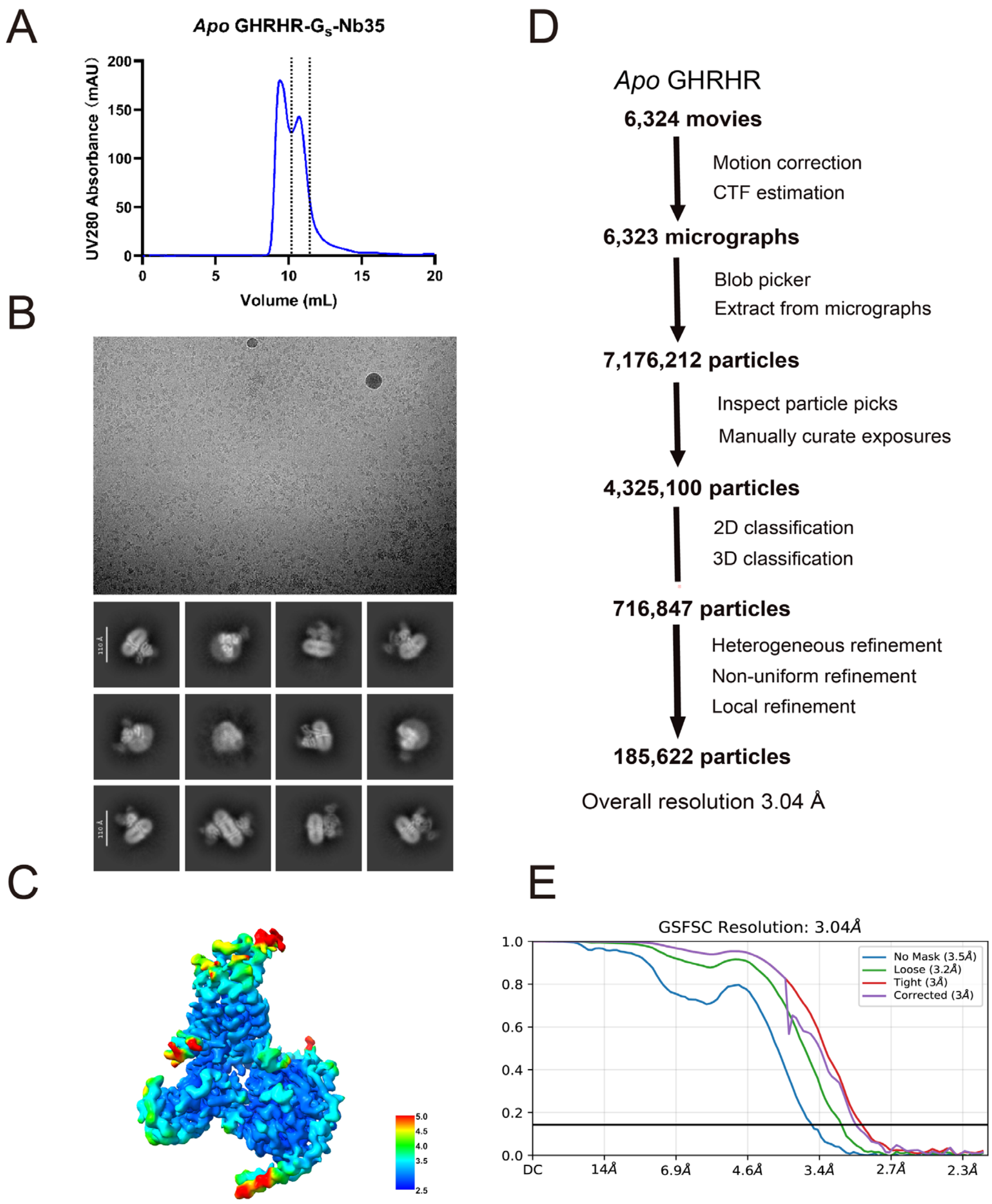

Figure S1. ***Apo*** **GHRHR–G$_s$ complex purification and cryo-EM data processing.** (**A**) Representative size-exclusion chromatography elution profile of *Apo* GHRHR–G$_s$ complex on Superdex 200 Increase 10/300 GL column. The monomer peak for the complex is indicated by dotted lines. (**B**) Representative cryo-EM micrograph and 2D classification of *Apo* GHRHR–G$_s$ complex. (**C**) 3D density map colored according to local resolution (Å). (**D**) The flowchart of cryo-EM data processing for *Apo* GHRHR–G$_s$ complex. Details can be found in Methods. (**E**) The "Gold standard" Fourier shell correlation (FSC) curve, indicating that the global resolution of the electron density map of the complex is 3.04 Å.

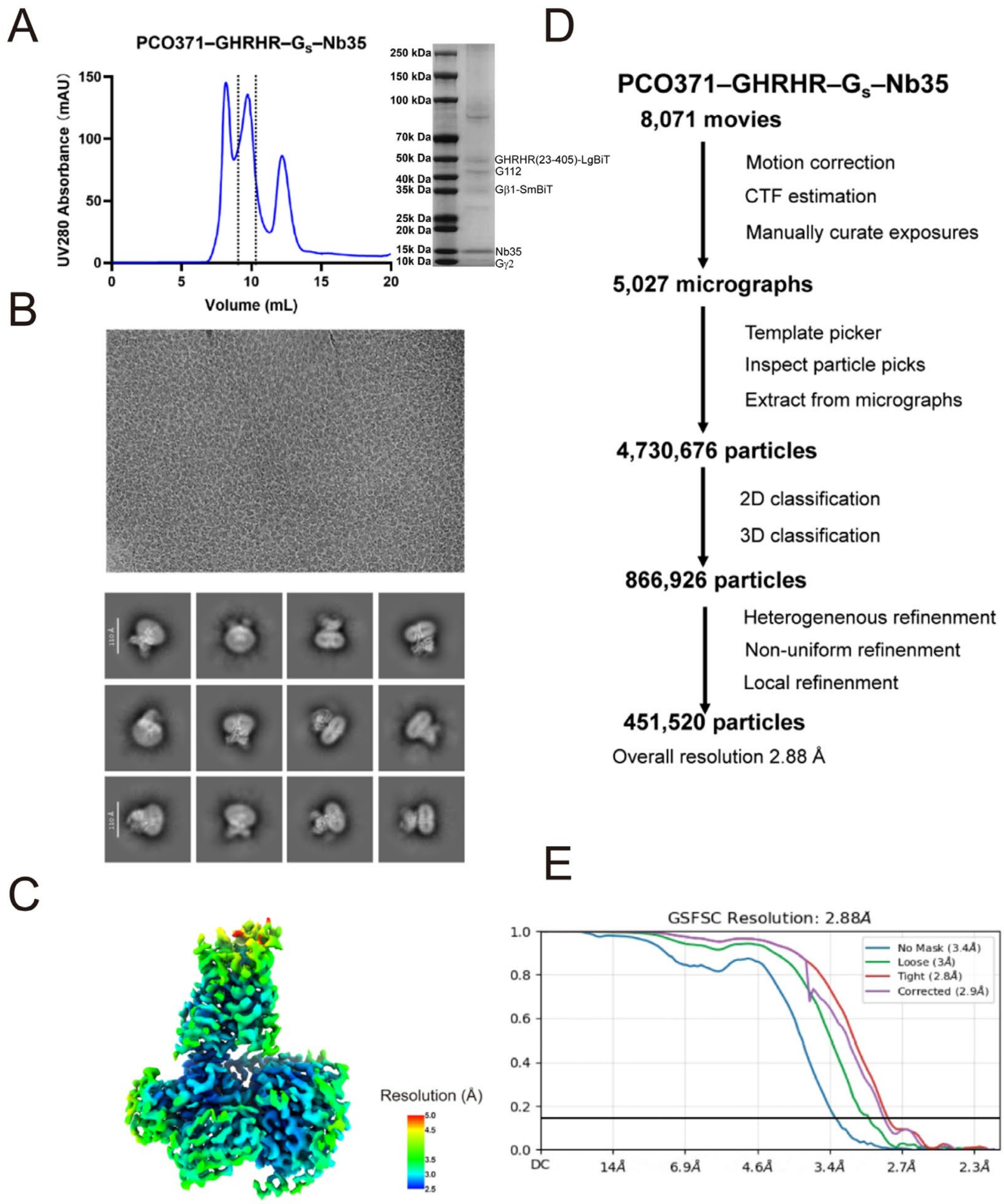

Figure S2. PCO371–GHRHR–G$_s$ complex purification and cryo-EM data processing. (**A**) Representative size-exclusion chromatography elution profile of PCO371–GHRHR–G$_s$ complex on Superdex 200 Increase 10/300 GL column. The monomer peak for the complex is indicated by dotted lines. (**B**) Representative cryo-EM micrograph and 2D classification of PCO371–GHRHR–G$_s$ complex. (**C**) 3D density map colored according to local resolution (Å). (**D**) The flowchart of cryo-EM data processing for PCO371–GHRHR–G$_s$ complex. Details can be found in Methods. (**E**) The "Gold standard" FSC curve, indicating that the global resolution of the electron density map of the complex is 2.88 Å.

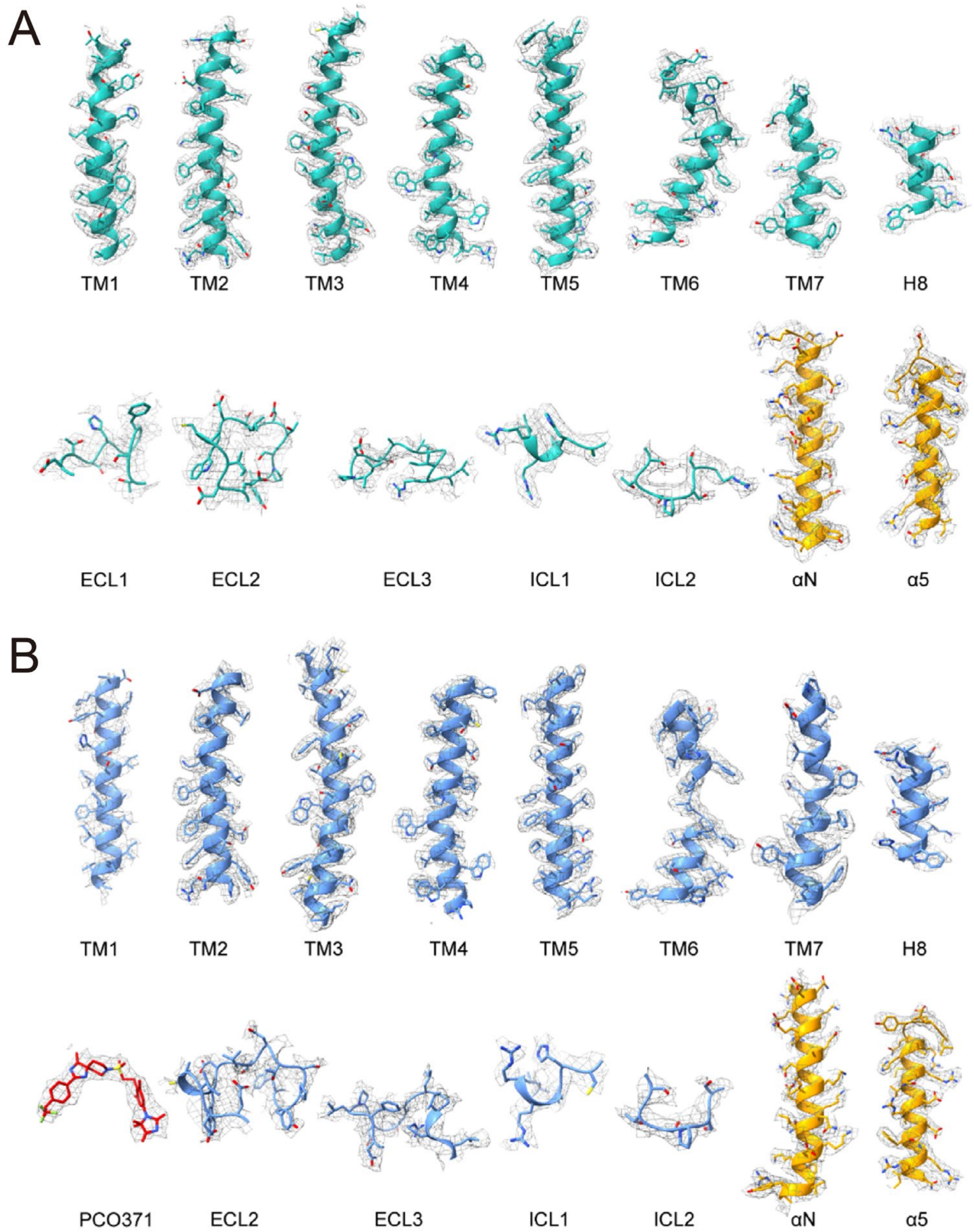

Figure S3. Near-atomic resolution models of the *Apo* GHRHR–G$_s$ and PCO371–GHRHR–G$_s$ complexes in the cryo-EM density maps. (**A**) EM density map and model of the *Apo* GHRHR–G$_s$ complex are shown for all seven transmembrane α-helices 1–7 (TMs 1–7), helix 8 (H8), extracellular loops 1–3 (ECLs 1–3), intracellular loops 1 and 2 (ICL1 and ICL2), and the α5-helix and αN-helix of Gα$_s$ subunit. (**B**) EM density map and model of the PCO371–GHRHR–G$_s$ complex are shown for TMs 1–7, H8, PCO371, ECL2, ECL3, ICL1, ICL2 and the α5-helix and αN-helix of Gα$_s$ subunit.

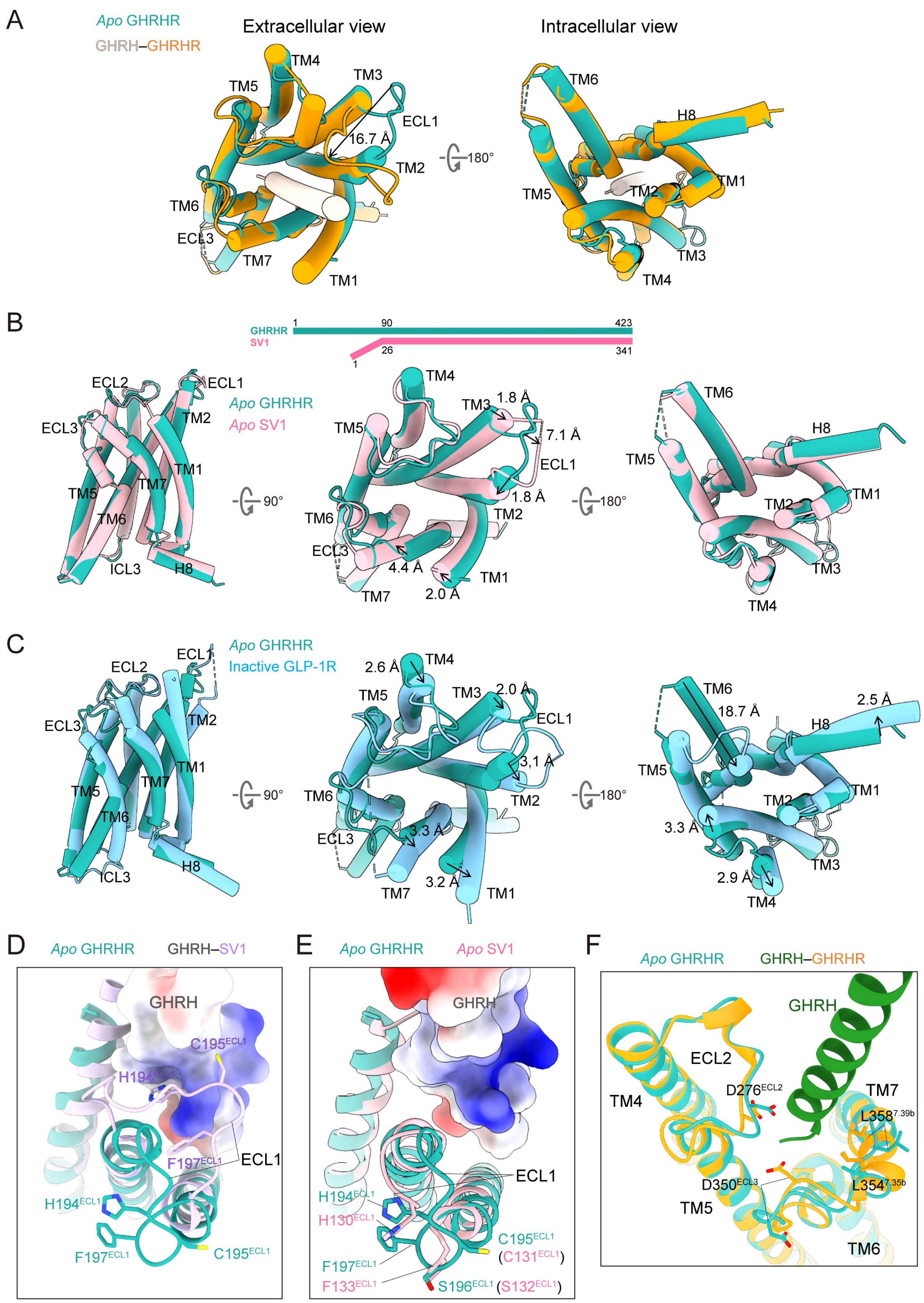

Figure S4. Comparison of the *Apo* GHRHR structure with that of GHRH-bound GHRHR, *Apo* SV1 and inactive state GLP-1R. (**A**) Superimposition of *Apo* and GHRH-bound GHRHR–G$_s$ (PDB: 7CZ5) complexes from extracellular (left) and intracellular (right) views, aligned on the receptor. The arrow indicates the movement of ECL1 measured by the Cα atoms of C195^{ECL1}. (**B**) Superimposed

TMDs of *Apo* GHRHR and *Apo* SV1 (PDB: 7V9L) from orthogonal (left), extracellular (middle) and intracellular (right) views. Black arrows indicate the movement of TM1, TM2, TM3, TM7 and ECL1 measured by the Cα atoms of T128$^{1.38b}$ (T64$^{1.38b}$ on SV1), S189^{ECL1} (S125^{ECL1} on SV1), V200$^{3.27b}$ (V136$^{3.27b}$ on SV1), P359$^{7.40b}$ (P295$^{7.40b}$ on SV1) and D193^{ECL1} (D129^{ECL1} on SV1), respectively. The notation below depicts the sequence variation of SV1 relative to wild-type (WT) GHRHR. (**C**) Superimposed TMDs of *Apo* GHRHR and inactive GLP-1R (PDB: 5VEW) from orthogonal (left), extracellular (middle) and intracellular (right) views. Black arrows indicate the extracellular movement of TM1, TM2, TM3, TM4 and TM7 measured by the Cα atoms of T128$^{1.38b}$ (F143$^{1.38b}$ on GLP-1R), A185$^{2.70b}$ (A200$^{2.70b}$ on GLP-1R), V200$^{3.27b}$ (L224$^{3.27b}$ on GLP-1R), F267$^{4.67b}$ (Y291$^{4.67b}$ on GLP-1R) and P359$^{7.40b}$ (F385$^{7.40b}$ on GLP-1R), as well as intracellular movement of TM3, TM4, TM6 and H8 measured by the Cα atoms of R240$^{4.40b}$ (W264$^{4.40b}$ on GLP-1R), A232$^{3.59b}$ (A256$^{3.59b}$ on GLP-1R), Q321$^{6.32b}$ (T343$^{6.32b}$ on GLP-1R) and W391$^{8.58b}$ (W417$^{8.58b}$ on GLP-1R), respectively. (**D**) Superposition of *Apo* GHRHR and GHRH-bound SV1, showing a similar peptide-induced inward movement of ECL1. (**E**) Superposition of *Apo* GHRHR and *Apo* SV1, illustrating the influence of ECD integrity on ECL1 conformations. Compared to the full-length ECD in GHRHR, the shorter ECD segment in *Apo* SV1 correlates with increased flexibility of ECL1, as evidenced by poor electron density for residues C131/S132 (equivalent to C195/S196 in GHRHR)(Rekasi et al., 2000). These two residues are well-defined in the *Apo* GHRHR structure. (**F**) Structural comparison between *Apo* and GHRH-bound GHRHR, highlighting conformational differences in ECL2, ECL3, and the extracellular segments of TMs 4-7. Key residues with notable rearrangements are shown as sticks and labeled with the class B1 GPCR numbering in superscript (Wootten et al., 2013).

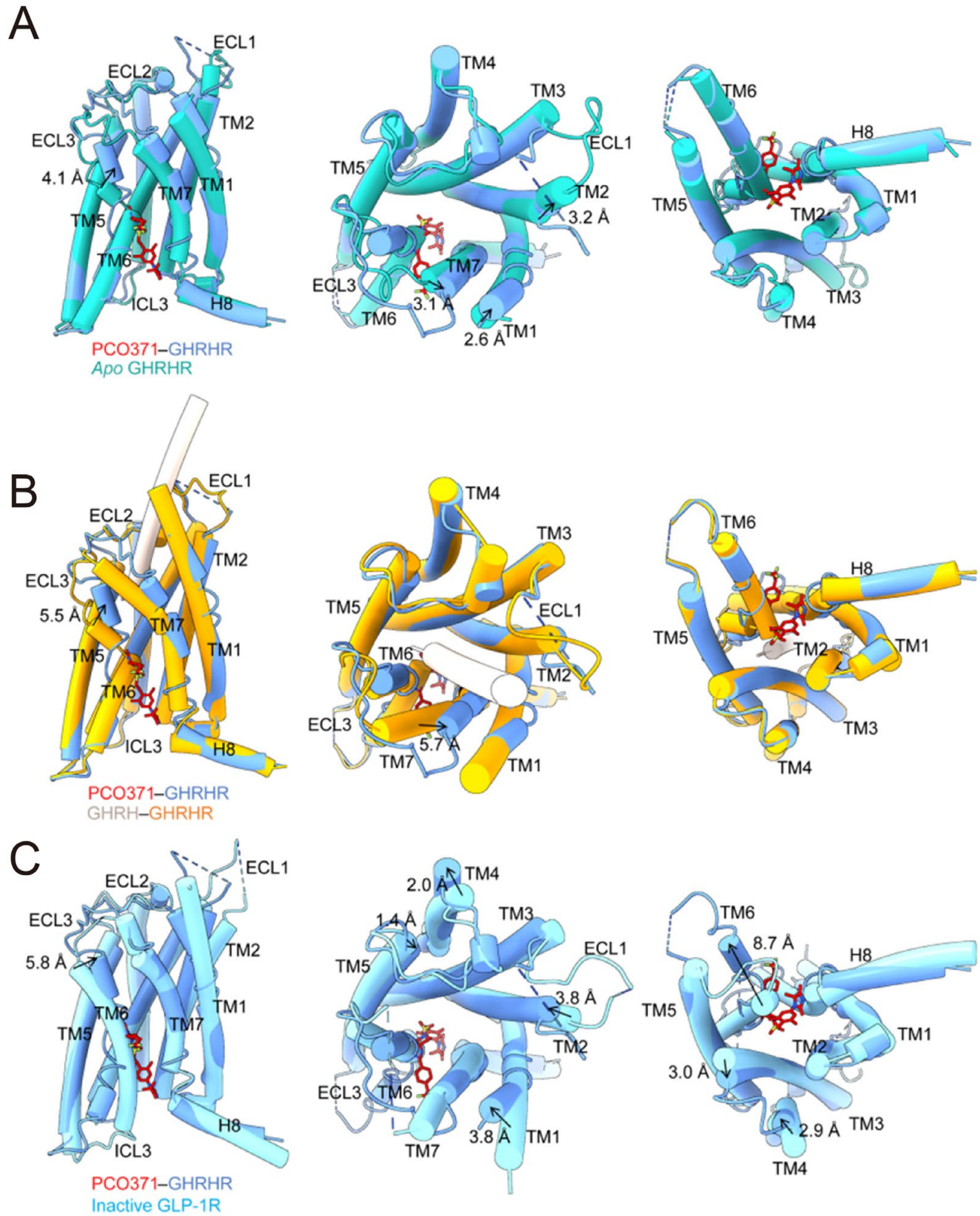

Figure S5. Structural comparison of PCO371-bound GHRHR with *Apo* GHRHR, GHRH-bound GHRHR and inactive GLP-1R. (**A**) Orthogonal (left), extracellular (middle), and intracellular (right) views of the *Apo* and PCO371-bound GHRHR–G_s complexes, aligned on the receptor TMDs. Arrows indicate conformational movements of TMs 1, 2, 6 and 7, measured by the displacement of Cα atoms at T128$^{1.38b}$, I186$^{2.71b}$, Y342$^{6.53b}$ and E361$^{7.42b}$, respectively. (**B**) Orthogonal (left), extracellular (middle), and intracellular (right) views comparing the TMDs of PCO371-bound and GHRH-bound GHRHR (PDB: 7CZ5). Black arrows indicate movements of TM6 and TM7, measured at the Cα atoms of I343$^{6.54b}$ and E361$^{7.42b}$, respectively. (**C**) Orthogonal (left), extracellular (middle), and intracellular (right) views comparing the TMDs of PCO371-bound GHRHR and inactive GLP-1R (PDB: 5VEW). Black arrows indicate extracellular movements of TMs 1, 2, 4, 5

and 6, measured by the Cα atoms of F126$^{1.36b}$ (L141$^{1.36b}$ on GLP-1R), A185$^{2.70b}$ (A200$^{2.70b}$ on GLP-1R), P267$^{4.67b}$ (Y291$^{4.67b}$ on GLP-1R), W283$^{5.37b}$ (L307$^{5.37b}$ on GLP-1R) and F345$^{6.56b}$ (F367$^{6.56b}$ on GLP-1R), respectively. Additional arrows indicate intracellular movements of TM3, TM4 and TM6, measured at Cα positions A232$^{3.59b}$ (A256$^{3.59b}$ on GLP-1R), R240$^{4.40b}$ (W264$^{4.40b}$ on GLP-1R) and R326$^{6.37b}$ (R348$^{6.37b}$ on GLP-1R), respectively.

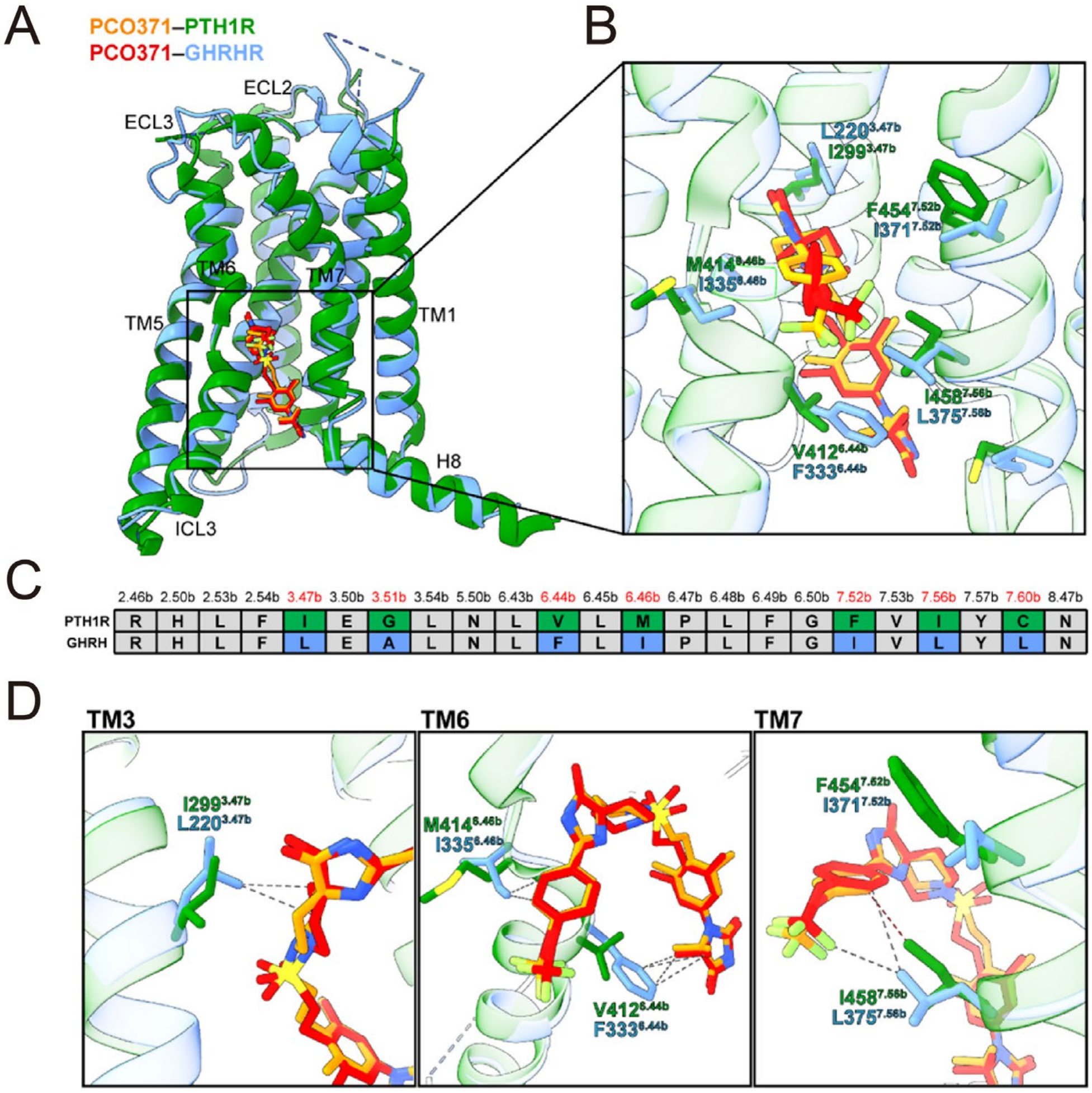

Figure S6. Comparison of the PCO371 binding pockets in GHRHR and PTH1R. (**A**) Structural superposition of the PCO371-bound GHRHR and PTH1R (PDB: 8JR9) complexes. (**B**) Close-up orthogonal view of the PCO371 binding sites in GHRHR and PTH1R. (**C**) Sequence alignment of representative residues interacting with PCO371 in GHRHR and PTH1R. Non-conserved residues are highlighted. (**D**) Comparison of key divergent residues on TM3 (left), TM6 (middle), and TM7 (right) involved in PCO371 binding to GHRHR versus PTH1R. PCO371 engages in more extensive contacts with the transmembrane bundle of GHRHR. Polar interactions are indicated by dashed lines (black for GHRHR; red for PTH1R).

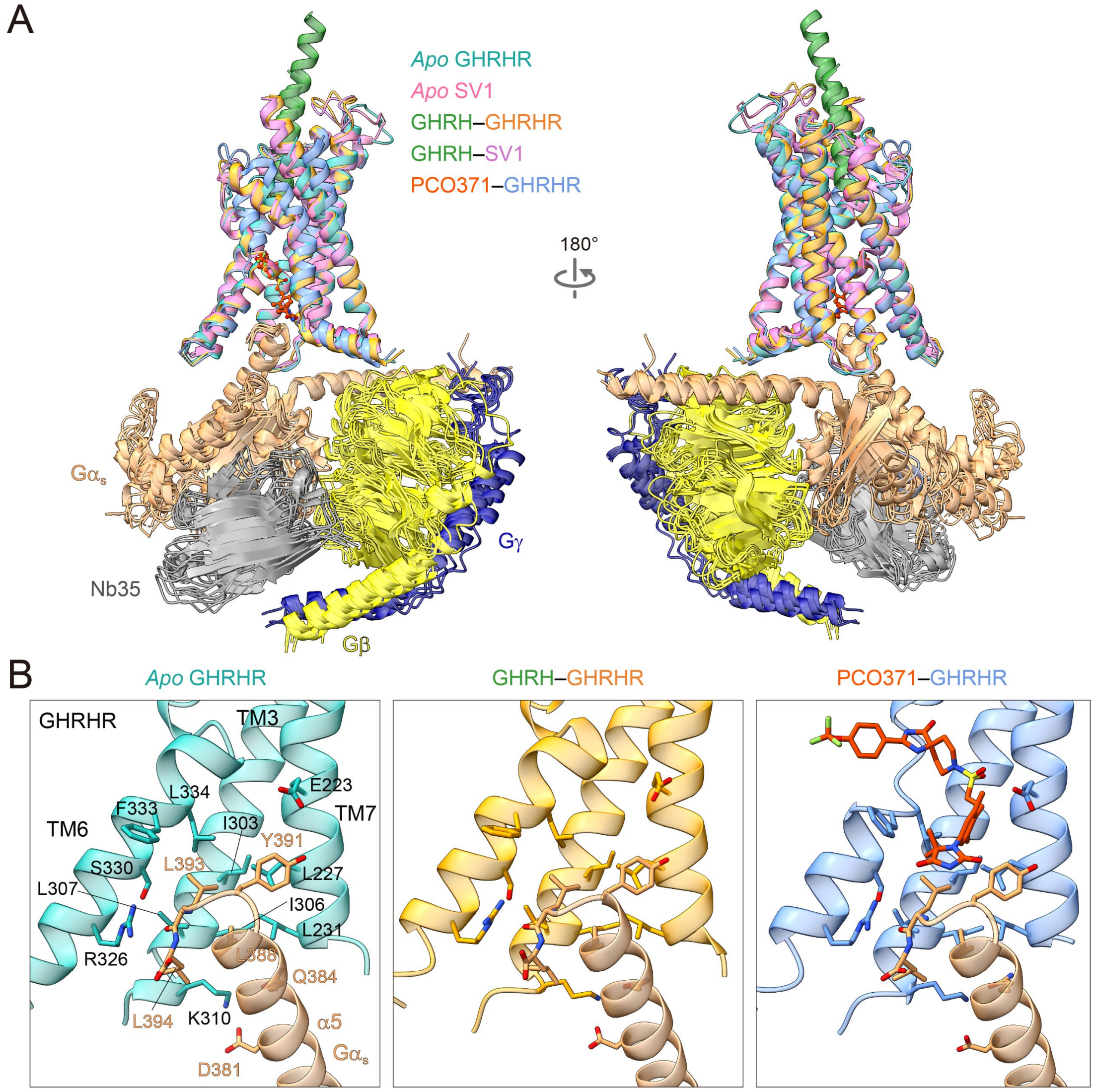

Figure S7. Structural comparison of G protein coupling across *Apo*, GHRH-bound, and PCO371-bound GHRHR structures. (**A**) Overall architecture of G protein coupling to GHRHR in the *Apo*, endogenous peptide (GHRH)-bound, and allosteric agonist (PCO371)-bound states, highlighting the conserved conformation for accommodating the G_s protein. The α5-helix of the $G\alpha_s$ Ras-like domain inserts into the intracellular crevice of the GHRHR TMD. (**B**) Comparison of interactions between TMs 3, 6, and 7 and the C-terminus of the $G\alpha_s$ α5-helix in the *Apo*, GHRH-bound, and PCO371-bound states. Key interacting residues are shown as sticks.

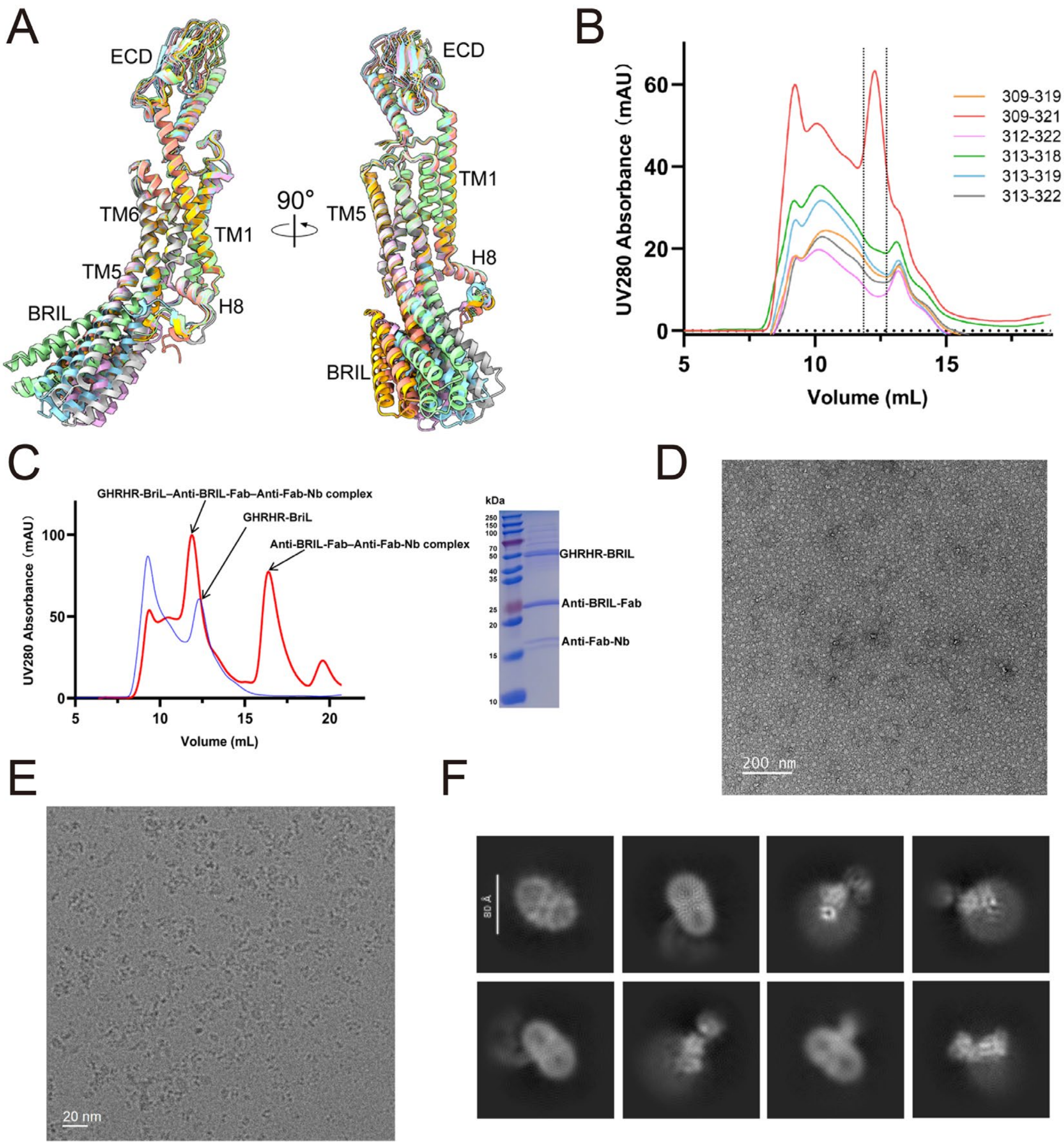

Figure S8. Biochemical and cryo-EM characterization of the inactive GHRHR–BRIL fusion construct. (**A**) Superposition of six representative predicted structural models of inactive GHRHR, highlighting the engineered BRIL domain insertion in ICL3. The BRIL domain and the receptor are connected through continuous helical structures. Continuous helical connections formed between the BRIL and the receptor. (**B**) SEC profiles (Superdex 200 Increase 10/300 GL column) for the six representative constructs shown in (A). The buffer contained 20 mM HEPES (pH 7.5), 100 mM NaCl, 25 μM TCEP, 0.00075% (w/v) LMNG, 0.00025% (w/v) GDN, and 0.00015% (w/v) CHS. Chromatograms were generated using GraphPad Prism. (**C**) SEC elution profile (left) and corresponding SDS-PAGE analysis (right) of the GHRHR-BRIL–anti-BRIL Fab–anti-Fab nanobody (Nb) complex. Peaks corresponding to the GHRHR-BRIL fusion protein and the final complex are indicated by arrows. The SEC profile was generated using GraphPad Prism. (**D**) Representative negative-staining EM micrograph of the GHRHR-BRIL–Anti-BRIL-Fab–Anti-Fab-Nb complex,

indicating high homogeneity of the sample. Scale bar, 200 nm. (**E**) Representative cryo-EM micrograph of the GHRHR-BRIL–Anti-BRIL-Fab–Anti-Fab-Nb complex. Scale bar, 20 nm. (**F**) Representative 2D class averages of the complex obtained by cryo-EM image processing. Scale bar, 80 Å. 2D, two-dimensional.

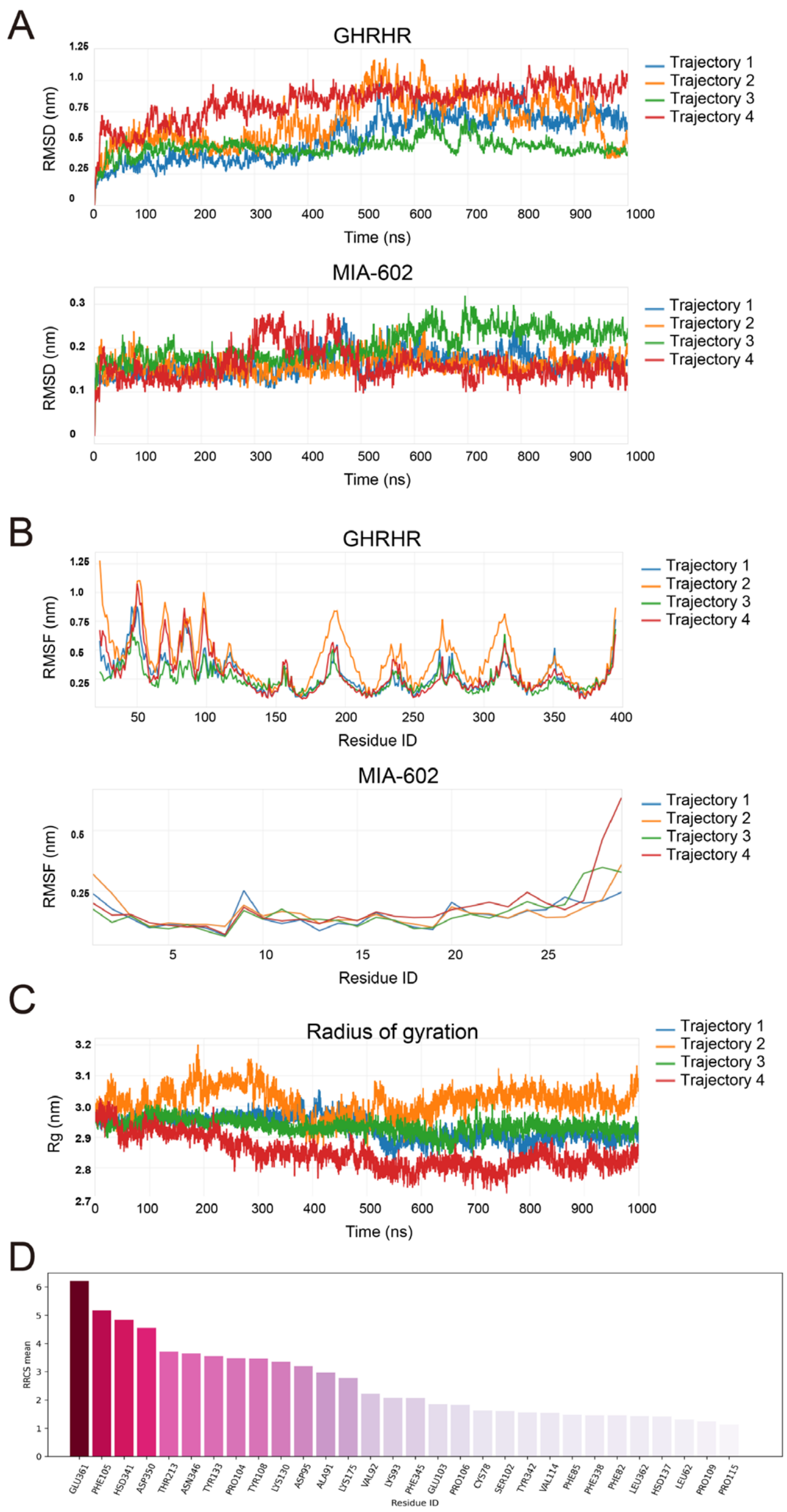

Figure S9. MD simulation of the antagonist MIA-602-bound GHRHR. (**A**) Time evolution of the backbone RMSD for the receptor (top) and the heavy-atom RMSD for the antagonist MIA-602 (bottom). (**B**) Per-residue root-mean-square fluctuation (RMSF) for the Cα atoms of GHRHR (top) and for the heavy atoms of MIA-602 (bottom). (**C**) Radius of gyration (Rg) of the simulated complexes over time. All analyses were performed on periodic boundary condition (PBC)-corrected trajectories after least-squares fitting to the initial structure; data from four independent replicates (Trajectory 1–4) are shown in distinct colors as indicated. (**D**) Ranked bar plot of the top 30 residue–residue contact scores (RRCS) quantifying MIA-602–GHRHR interactions over the final 500 ns of simulation. The steep decline in scores beyond the top few residues indicates that binding is primarily stabilized by a compact, persistent interaction core.

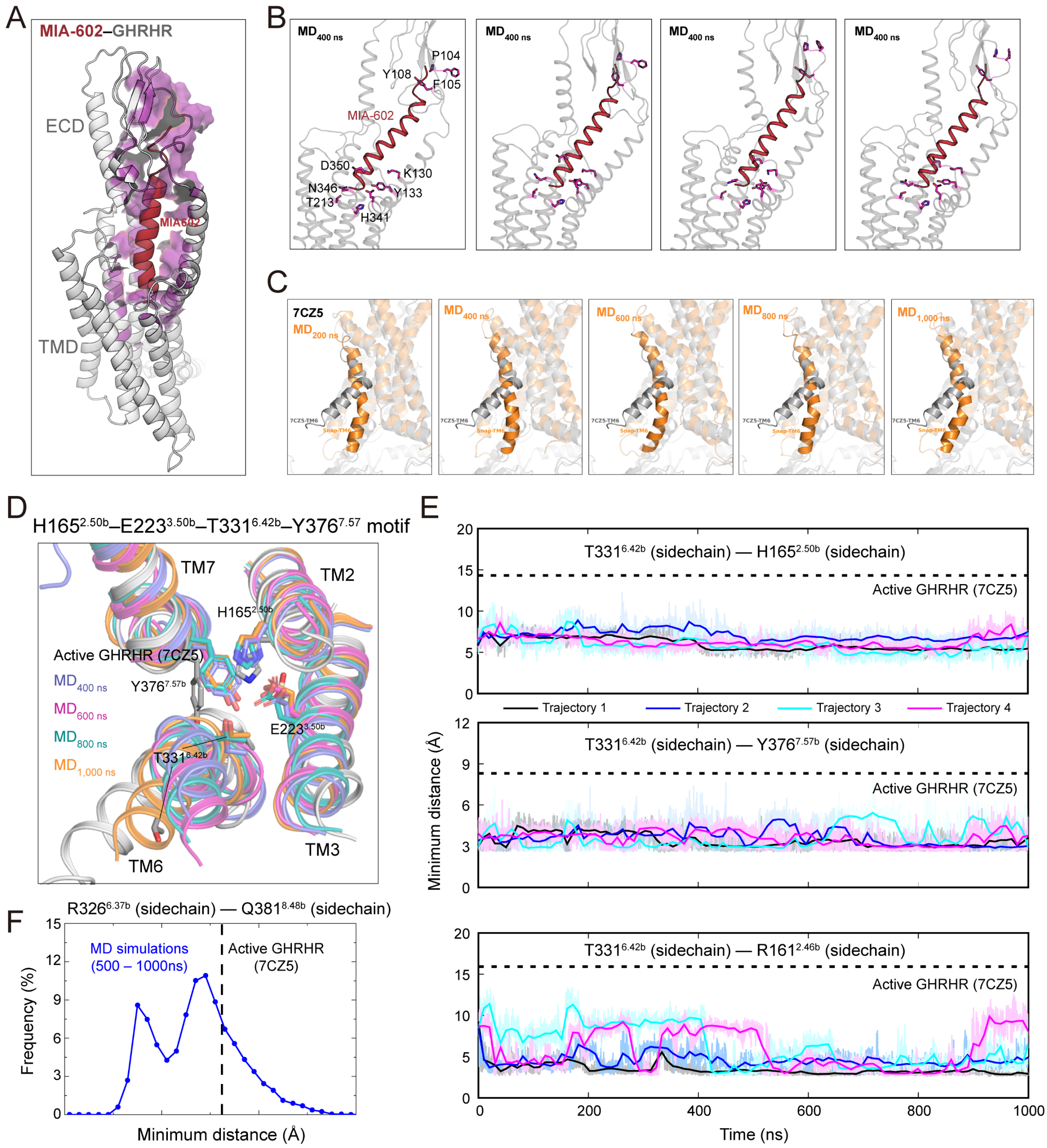

Figure S10. Molecular dynamics investigation of antagonist-bound GHRHR. (**A**) Overall structure of GHRHR bound to the antagonist MIA-602. The simulation system was built from an inactive GHRHR model (AlphaFold2-Multistate), embedded in a lipid bilayer, solvated with explicit TIP3P water, and subjected to all-atom MD simulations. (**B**) Ligand-binding pocket in representative MD snapshots (600, 800 and 1000 ns) of the MIA-602-bound GHRHR. (**C**) Conformation of TM6 in the MIA-602-bound state (representative MD snapshots) compared to that in the active GHRHR (cryo-EM structure). (**D**) Conformation of the H165$^{2.50b}$–E223$^{3.50b}$–T331$^{6.42b}$–Y376$^{7.57b}$ (HETY) motif in the antagonist-bound state (representative MD snapshots) compared to that in the active GHRHR (cryo-EM). (**E**) Time evolution of the minimum distances between the intracellular half of TM6 and key residues on TM3/TM7 during the MD simulation. The significantly larger distances in

the active, G_s-coupled state (PDB: 7CZ5; dashed line) reflect the outward movement of TM6 required for G-protein coupling. The thick and thin traces represent moving averages and original, unsmoothed values, respectively. (**F**) Distribution of the minimum distance between R326$^{6.37b}$ and Q381$^{8.48b}$ calculated from the last 500 ns of four independent MD trajectories. The corresponding distance in the active structure (PDB: 7CZ5) is indicated by a dashed line for reference.

Table S1. Cryo-EM data collection, model refinement and validation statistics.

	Apo GHRHR–G_s	PCO371–GHRHR–G_s
PDB code	24TB	24TI
EMDB code	EMD-69803	EMD-69804
**Data collection and processing**		
Magnification	105,000	105,000
Voltage (kV)	300	300
Electron exposure ($e^-/Å^2$)	80	80
Defocus range (μm)	−1.2 to −2.2	−1.2 to −2.2
Pixel size (Å)	1.071	1.071
Symmetry imposed	C1	C1
Initial particle projections (no.)	7,176,212	4,730,676
Final particle projections (no.)	185,622	451,520
Map resolution (Å)	3.04	2.88
FSC threshold	0.143	0.143
Map resolution range (Å)	2.77−3.26	2.50−3.08
Map sharpening *B* factor ($Å^2$)	−102.8	-112.8
**Refinement**		
Initial model used (PDB code)	7CZ5	7CZ5
Model resolution (Å)	3.12	2.90
FSC threshold	0.143	0.143
Model composition		
Chains	4	6
Non-hydrogen atoms	6,975	8,027
Protein residues	879	1,006
Water	0	0
Ligands	0	1
RMSD		
Bond lengths (Å)	0.004	0.004

Bond angles (°)	0.514	0.487
Validation		
MolProbity score	2.02	1.72
Clashscore	6.63	6.28
Rotamer outliers (%)	2.52	2.57
Ramachandran plot		
Favored (%)	94.90	95.44
Allowed (%)	5.10	4.26
Disallowed (%)	0	0.3

RMSD, root-mean-square deviation.

References

ADAMS, P. D., AFONINE, P. V., BUNKOCZI, G., CHEN, V. B., DAVIS, I. W., ECHOLS, N., HEADD, J. J., HUNG, L. W., KAPRAL, G. J., GROSSE-KUNSTLEVE, R. W., MCCOY, A. J., MORIARTY, N. W., OEFFNER, R., READ, R. J., RICHARDSON, D. C., RICHARDSON, J. S., TERWILLIGER, T. C. & ZWART, P. H. 2010. PHENIX: a comprehensive Python-based system for macromolecular structure solution. *Acta Crystallogr D Biol Crystallogr,* 66**,** 213-21.

CARPENTER, B., NEHME, R., WARNE, T., LESLIE, A. G. & TATE, C. G. 2016. Structure of the adenosine A(2A) receptor bound to an engineered G protein. *Nature,* 536**,** 104-7.

CARY, B. P., ZHANG, X., CAO, J., JOHNSON, R. M., PIPER, S. J., GERRARD, E. J., WOOTTEN, D. & SEXTON, P. M. 2023. New insights into the structure and function of class B1 GPCRs. *Endocr Rev,* 44**,** 492-517.

CHEN, H., HUANG, W. & LI, X. 2022. Structures of oxysterol sensor EBI2/GPR183, a key regulator of the immune response. *Structure,* 30**,** 1016-1024 e5.

CONG, Z., CHEN, L. N., MA, H., ZHOU, Q., ZOU, X., YE, C., DAI, A., LIU, Q., HUANG, W., SUN, X., WANG, X., XU, P., ZHAO, L., XIA, T., ZHONG, W., YANG, D., ERIC XU, H., ZHANG, Y. & WANG, M. W. 2021a. Molecular insights into ago-allosteric modulation of the human glucagon-like peptide-1 receptor. *Nat Commun,* 12**,** 3763.

CONG, Z., ZHAO, F., LI, Y., LUO, G., MAI, Y., CHEN, X., CHEN, Y., LIN, S., CAI, X., ZHOU, Q., YANG, D. & WANG, M.-W. 2024. Molecular features of the ligand-free GLP-1R, GCGR and GIPR in complex with G(s) proteins. *Cell Discov,* 10**,** 18.

CONG, Z., ZHOU, F., ZHANG, C., ZOU, X., ZHANG, H., WANG, Y., ZHOU, Q., CAI, X., LIU, Q., LI, J., SHAO, L., MAO, C., WANG, X., WU, J., XIA, T., ZHAO, L. H., JIANG, H., ZHANG, Y., XU, H. E., CHENG, X., YANG, D. & WANG, M.-W. 2021b. Constitutive signal bias mediated by the human GHRHR splice variant 1. *Proc Natl Acad Sci U S A,* 118**,** e2106606118.

DIXON, A. S., SCHWINN, M. K., HALL, M. P., ZIMMERMAN, K., OTTO, P., LUBBEN, T. H., BUTLER, B. L., BINKOWSKI, B. F., MACHLEIDT, T., KIRKLAND, T. A., WOOD, M. G., EGGERS, C. T., ENCELL, L. P. & WOOD, K. V. 2016. NanoLuc complementation reporter optimized for accurate measurement of protein interactions in cells. *ACS Chem Biol,* 11**,** 400-8.

DUAN, J., SHEN, D. D., ZHOU, X. E., BI, P., LIU, Q. F., TAN, Y. X., ZHUANG, Y. W., ZHANG, H. B., XU, P. Y., HUANG, S. J., MA, S. S., HE, X. H., MELCHER, K., ZHANG, Y., XU, H. E. & JIANG, Y. 2020. Cryo-EM structure of an activated VIP1 receptor-G protein complex revealed by a NanoBiT tethering strategy. *Nat Commun,* 11**,** 4121.

EMSLEY, P. & COWTAN, K. 2004. Coot: model-building tools for molecular graphics. *Acta Crystallogr D Biol Crystallogr,* 60**,** 2126-32.

FAHRENHOLTZ, C. D., RICK, F. G., GARCIA, M. I., ZARANDI, M., CAI, R. Z., BLOCK, N. L., SCHALLY, A. V. & BURNSTEIN, K. L. 2014. Preclinical efficacy of growth hormone-releasing hormone antagonists for androgen-dependent and castration-resistant human prostate cancer. *Proc Natl Acad Sci U S A,* 111**,** 1084-9.

GESMUNDO, I., PEDROLLI, F., CAI, R., SHA, W., SCHALLY, A. V. & GRANATA, R. 2025. Growth hormone-releasing hormone and cancer. *Rev Endocr Metab Disord,* 26**,** 443-456.

GRANATA, R., LEONE, S., ZHANG, X., GESMUNDO, I., STEENBLOCK, C., CAI, R., SHA, W., GHIGO, E., HARE, J. M., BORNSTEIN, S. R. & SCHALLY, A. V. 2025. Growth hormone-releasing hormone and its analogues in health and disease. *Nat Rev Endocrinol,* 21**,** 180-195.

GUO, Q., HE, B., ZHONG, Y., JIAO, H., REN, Y., WANG, Q., GE, Q., GAO, Y., LIU, X., DU, Y., HU, H. & TAO, Y. 2024. A method for structure determination of GPCRs in various states. *Nat Chem Biol,* 20**,** 74-82.

HAN, W., CHEN, Z., WANG, M.-W. & ZHOU, Q. 2025. gmx_RRCS: a precision tool for detecting subtle conformational dynamics in molecular simulations. *J Mol Biol,* 437**,** 169129.

HUANG, J., RAUSCHER, S., NAWROCKI, G., RAN, T., FEIG, M., DE GROOT, B. L., GRUBMULLER, H. & MACKERELL, A. D., JR. 2017. CHARMM36m: an improved force field for folded and intrinsically disordered proteins. *Nat Methods,* 14**,** 71-73.

KOBAYASHI, K., KAWAKAMI, K., KUSAKIZAKO, T., TOMITA, A., NISHIMURA, M., SAWADA, K., OKAMOTO, H. H., HIRATSUKA, S., NAKAMURA, G., KUWABARA, R., NODA, H., MURAMATSU, H., SHIMIZU, M., TAGUCHI, T., INOUE, A., MURATA, T. & NUREKI, O. 2023. Class B1 GPCR activation by an intracellular agonist. *Nature,* 618**,** 1085-1093.

LEE, J., CHENG, X., SWAILS, J. M., YEOM, M. S., EASTMAN, P. K., LEMKUL, J. A., WEI, S., BUCKNER, J., JEONG, J. C., QI, Y., JO, S., PANDE, V. S., CASE, D. A., BROOKS, C. L., 3RD, MACKERELL, A. D., JR., KLAUDA, J. B. & IM, W. 2016. CHARMM-GUI input generator for NAMD, GROMACS, AMBER, OpenMM, and CHARMM/OpenMM simulations using the CHARMM36 additive force Field. *J Chem Theory Comput,* 12**,** 405-13.

LI, W., ZHOU, Q., CONG, Z., YUAN, Q., LI, W., ZHAO, F., XU, H. E., ZHAO, L. H., YANG, D. & WANG, M.-W. 2024. Structural insights into the triple agonism at GLP-1R, GIPR and GCGR manifested by retatrutide. *Cell Discov,* 10**,** 77.

MASTRONARDE, D. N. 2005. Automated electron microscope tomography using robust prediction of specimen movements. *J Struct Biol,* 152**,** 36-51.

PANDY-SZEKERES, G., CAROLI, J., MAMYRBEKOV, A., KERMANI, A. A., KESERU, G. M., KOOISTRA, A. J. & GLORIAM, D. E. 2023. GPCRdb in 2023: state-specific structure models using AlphaFold2 and new ligand resources. *Nucleic Acids Res,* 51**,** D395-D402.

PEREZ, R., SCHALLY, A. V., VIDAURRE, I., RINCON, R., BLOCK, N. L. & RICK, F. G. 2012. Antagonists of growth hormone-releasing hormone suppress in vivo tumor growth and gene expression in triple negative breast cancers. *Oncotarget,* 3**,** 988-97.

PETTERSEN, E. F., GODDARD, T. D., HUANG, C. C., COUCH, G. S., GREENBLATT, D. M., MENG, E. C. & FERRIN, T. E. 2004. UCSF Chimera--a visualization system for exploratory research and analysis. *J Comput Chem,* 25**,** 1605-12.

PETTERSEN, E. F., GODDARD, T. D., HUANG, C. C., MENG, E. C., COUCH, G. S., CROLL, T. I., MORRIS, J. H. & FERRIN, T. E. 2021. UCSF ChimeraX: Structure visualization for researchers, educators, and developers. *Protein Sci,* 30**,** 70-82.

PUNJANI, A., RUBINSTEIN, J. L., FLEET, D. J. & BRUBAKER, M. A. 2017. cryoSPARC: algorithms for rapid unsupervised cryo-EM structure determination. *Nat Methods,* 14**,** 290-296.

REKASI, Z., CZOMPOLY, T., SCHALLY, A. V. & HALMOS, G. 2000. Isolation and sequencing of cDNAs for splice variants of growth hormone-releasing hormone receptors from human cancers. *Proc Natl Acad Sci U S A,* 97**,** 10561-6.

SONG, G., YANG, D., WANG, Y., DE GRAAF, C., ZHOU, Q., JIANG, S., LIU, K., CAI, X., DAI, A., LIN, G., LIU, D., WU, F., WU, Y., ZHAO, S., YE, L., HAN, G. W., LAU, J., WU, B., HANSON, M. A., LIU, Z. J., WANG, M.-W. & STEVENS, R. C. 2017. Human GLP-1 receptor transmembrane domain structure in complex with allosteric modulators. *Nature,* 546**,** 312-315.

STANLEY, T. L., FELDPAUSCH, M. N., OH, J., BRANCH, K. L., LEE, H., TORRIANI, M. & GRINSPOON, S. K. 2014. Effect of tesamorelin on visceral fat and liver fat in HIV-infected patients with abdominal fat accumulation: a randomized clinical trial. *JAMA,* 312**,** 380-9.

SUN, W., CHEN, L. N., ZHOU, Q., ZHAO, L. H., YANG, D., ZHANG, H., CONG, Z., SHEN, D. D., ZHAO, F., ZHOU, F., CAI, X., CHEN, Y., ZHOU, Y., GADGAARD, S., VAN DER VELDEN, W. J. C., ZHAO, S., JIANG, Y., ROSENKILDE, M. M., XU, H. E., ZHANG, Y. & WANG, M.-W. 2020. A unique hormonal recognition feature of the human glucagon-like peptide-2 receptor. *Cell Res,* 30**,** 1098-1108.

TAMURA, T., NODA, H., JOYASHIKI, E., HOSHINO, M., WATANABE, T., KINOSAKI, M., NISHIMURA, Y., ESAKI, T., OGAWA, K., MIYAKE, T., ARAI, S., SHIMIZU, M., KITAMURA, H., SATO, H. & KAWABE, Y. 2016. Identification of an orally active small-molecule PTHR1 agonist for the treatment of hypoparathyroidism. *Nat Commun,* 7**,** 13384.

WOOTTEN, D., SIMMS, J., MILLER, L. J., CHRISTOPOULOS, A. & SEXTON, P. M. 2013. Polar transmembrane interactions drive formation of ligand-specific and signal pathway-biased family B G protein-coupled receptor conformations. *Proc Natl Acad Sci U S A,* 110**,** 5211-6.

YANG, D., ZHOU, Q., LABROSKA, V., QIN, S., DARBALAEI, S., WU, Y., YULIANTIE, E., XIE, L., TAO, H., CHENG, J., LIU, Q., ZHAO, S., SHUI, W., JIANG, Y. & WANG, M.-W. 2021. G protein-coupled receptors: structure- and function-based drug discovery. *Signal Transduct Target Ther,* 6**,** 7.

ZHANG, K., WU, H., HOPPE, N., MANGLIK, A. & CHENG, Y. 2022. Fusion protein strategies for cryo-EM study of G protein-coupled receptors. *Nat Commun,* 13**,** 4366.

ZHANG, Y., SUN, B., FENG, D., HU, H., CHU, M., QU, Q., TARRASCH, J. T., LI, S., SUN KOBILKA, T., KOBILKA, B. K. & SKINIOTIS, G. 2017. Cryo-EM structure of the activated GLP-1 receptor in complex with a G protein. *Nature,* 546**,** 248-253.

ZHAO, F., ZHANG, C., ZHOU, Q., HANG, K., ZOU, X., CHEN, Y., WU, F., RAO, Q., DAI, A., YIN, W., SHEN, D. D., ZHANG, Y., XIA, T., STEVENS, R. C., XU, H. E., YANG, D., ZHAO, L. & WANG, M.-W. 2021. Structural insights into hormone recognition by the human glucose-dependent insulinotropic polypeptide receptor. *Elife,* 10.

ZHAO, L. H., HE, Q., YUAN, Q., GU, Y., HE, X., SHAN, H., LI, J., WANG, K., LI, Y., HU, W., WU, K., SHEN, J. & XU, H. E. 2023. Conserved class B GPCR activation by a biased intracellular agonist. *Nature,* 621**,** 635-641.

ZHENG, S. Q., PALOVCAK, E., ARMACHE, J. P., VERBA, K. A., CHENG, Y. & AGARD, D. A. 2017. MotionCor2: anisotropic correction of beam-induced motion for improved cryo-electron microscopy. *Nat Methods,* 14**,** 331-332.

ZHOU, F., YE, C., MA, X., YIN, W., CROLL, T. I., ZHOU, Q., HE, X., ZHANG, X., YANG, D., WANG, P., XU, H. E., WANG, M.-W. & JIANG, Y. 2021. Molecular basis of ligand recognition and activation of human V2 vasopressin receptor. *Cell Res,* 31**,** 929-931.

ZHOU, F., ZHANG, H., CONG, Z., ZHAO, L. H., ZHOU, Q., MAO, C., CHENG, X., SHEN, D. D., CAI, X., MA, C., WANG, Y., DAI, A., ZHOU, Y., SUN, W., ZHAO, F., ZHAO, S., JIANG, H., JIANG, Y., YANG, D., ERIC XU, H., ZHANG, Y. & WANG, M.-W. 2020. Structural basis for activation of the growth hormone-releasing hormone receptor. *Nat Commun,* 11**,** 5205.

ZHOU, Q., YANG, D., WU, M., GUO, Y., GUO, W., ZHONG, L., CAI, X., DAI, A., JANG, W., SHAKHNOVICH, E. I., LIU, Z. J., STEVENS, R. C., LAMBERT, N. A., BABU, M. M., WANG, M.-W. & ZHAO, S. 2019. Common activation mechanism of class A GPCRs. *Elife,* 8**,** e50279.
